# City-level livestock methane emissions in China from 2010 to 2020

**DOI:** 10.1038/s41597-024-03072-y

**Published:** 2024-02-28

**Authors:** Mingxi Du, Xiang Kang, Qiuyu Liu, Haifeng Du, Jianjun Zhang, Yulong Yin, Zhenling Cui

**Affiliations:** 1 School of Public Policy and Administration, Xi’an Jiaotong University, Xi’an, 710049 China; 2 School of Land Science and Technology, China University of Geosciences, Beijing, 100083 China; 3 College of Resources and Environmental Sciences, National Academy of Agriculture Green Development, Key Laboratory of Plant-Soil Interactions, Ministry of Education, China Agricultural University, Beijing, 100193 China

**Keywords:** Environmental social sciences, Environmental sciences

## Abstract

Livestock constitute the world’s largest anthropogenic source of methane (CH_4_), providing high-protein food to humans but also causing notable climate risks. With rapid urbanization and increasing income levels in China, the livestock sector will face even higher emission pressures, which could jeopardize China’s carbon neutrality target. To formulate targeted methane reduction measures, it is crucial to estimate historical and current emissions on fine geographical scales, considering the high spatial heterogeneity and temporal variability of livestock emissions. However, there is currently a lack of time-series data on city-level livestock methane emissions in China, despite the flourishing livestock industry and large amount of meat consumed. In this study, we constructed a city-level livestock methane emission inventory with dynamic spatial-temporal emission factors considering biological, management, and environmental factors from 2010 to 2020 in China. This inventory could serve as a basic database for related research and future methane mitigation policy formulation, given the population boom and dietary changes.

## Background & Summary

Methane is the second most important greenhouse gas (GHG) and has a high global warming potential (GWP) 28–36 times that of carbon dioxide (CO_2_) over a 100-year period^1^. The livestock sector is the largest global emitter of anthropogenic methane, accounting for approximately one-third of emissions^2,3^. Among all the nations, China is the world’s largest anthropogenic methane emitter, with approximately 48 (37.5–61.7) Tg emitted in 2019, with the livestock sector ranking second^4^. As urbanization and income levels improve, the demand for animal-derived foods such as beef, milk, mutton, and eggs is projected to rapidly increase, leading to a significant increase in methane emissions and higher methane mitigation pressure in the future^4–7^. The development of a comprehensive, fine-scaled livestock methane inventory is crucial for analyzing historical emission trends and formulating regional mitigation plans^8^. While some studies have focused on addressing China’s livestock methane emissions, significant challenges remain in providing more robust and detailed inventory datasets^9–11^.

China’s administrative system generally encompasses five components, including province-, prefecture-, county-, and town-level administrative units. Prefecture-level administrative units include cities and autonomous regions, which explains the reference to a city-level emission inventory in this study because most of them are prefecture-level cities (84%). Prefecture-level cities (autonomous regions) are among the basic administrative units in China, highlighting the necessity of city-level analysis in GHG mitigation^12,13^. Due to the significant spatial differences in socioeconomic conditions and GHG emission patterns in China, fine geographical unit-based targeted methane mitigation actions are needed to achieve carbon neutrality^14,15^. To our knowledge, long-term series methane emission inventories at the city level are rare, with most studies conducted at the national or provincial level^12,16,17^. Although several studies have yielded grid-level livestock methane emission results for certain years, most studies are only based on the Gridded Livestock of the World (GLW) dataset with livestock density information^11,18,19^; these studies exhibit high uncertainties relative to China’s actual livestock status, which could be amplified in long-term series inventory construction. Indeed, significant socioeconomic differences exist even within cities, rendering city-level analysis necessary for mitigation and research purposes^9,20^. The development of methane inventories considering fine geographical units such as cities could provide promising applications, as agricultural production, including livestock production, is highly sensitive to spatial location and socioeconomic differences^21–23^.

Moreover, the analysis of historical emission trends is crucial for comprehensive mitigation studies, providing valuable information not only for greenhouse gas mitigation but also for related studies such as atmospheric environment simulation and agricultural green development^24,25^. Considering that factors such as high urbanization and increasing income levels have resulted in dietary transitions, these factors have impacted methane emissions due to changes in livestock category structures and animal populations^6,26,27^. Understanding temporal emission trends, particularly those of emissions originating from different livestock categories and geographical units, is essential for formulating effective mitigation strategies that account for different variations. Analyzing temporal emission trends at the city level could provide a foundation for policy evaluation, dietary transition studies and climate simulations. Time-series datasets offer more detailed information than cross-sectional data when examining temporal variations, predicting future trends, performing integrative analysis with socioeconomic development, etc. Here records of prefecture-level city livestock methane emissions could bridge the gaps in China’s methane data with finer spatial units and long time-series emissions. The data could be used for accurate mitigation policy development, future emission scenario analysis, spatial-temporal emission characteristic determination, and livestock industry efficiency evaluation. Additionally, our dataset provides information on the link between agriculture and climate change, which facilitates interdisciplinary study.

In general, GHG emissions were estimated by multiplying activity data and emission factors. Activity data provide a quantitative measure of the anthropogenic activities that emit GHGs, such as the amount of cement produced, coal burned, and fertilizer applied. The emission factor denotes the GHG emissions per unit of activity. Regarding livestock methane emissions, activity data include the animal population in a given year, and the EF indicates the methane emissions per animal head. Livestock methane emission estimation heavily depends on emission factors (EFs), which also exhibit spatial-temporal dynamic characteristics resulting from socioeconomic and agricultural technological developments^10,28^. Despite the significant progress achieved in previous studies on livestock methane emissions, spatial differences in key factors, such as body weight, productive performance (e.g., milk yield), manure management system, temperature and age structure, which are all closely associated with EFs, have not been comprehensively considered or even estimated using constant EFs^17,29–31^.

For instance, cattle in northern China are significantly heavier than those in southern China, resulting in obvious gross energy differences in EF calculation^11^. From a manure management perspective, certain management practices, such as daily spreading and anaerobic digestion, may result in lower methane emissions in regions due to the lower extent of the anaerobic environment or high recycling rate^10,32^. Additionally, these animal characteristics and management systems vary regionally and temporally, further emphasizing the need to transition from traditional constant EFs to spatial-temporal dynamic EFs to improve the estimation accuracy and reflect additional emission details. The key issue in the determination of dynamic EFs is the consideration of spatial and temporal conditions (such as biological, management, and environmental factors) comprehensively and simultaneously, and the parameters used in our study must include differentiated regional features. In terms of activity data, the age and sex of the slaughtered animal population and the age and structure are also key factors that may affect the amount and structure of the data. City-level activity data with detailed animal category information and structural information could decrease uncertainty and reveal detailed emission structures for further research.

Here, we constructed a long-term series of city-level livestock methane emissions over the past decade in China encompassing more than 340 prefecture-level administrative units, using city-level activity data and spatial-temporal dynamic EFs. City-level activity data were obtained from city statistical yearbooks, provincial statistical yearbooks and official reports (such as the National Economic and Social Development Bulletin). The temporal and regional variations in EFs were estimated by comprehensively considering the actual animal production and physical characteristics, including animal body weight, milk fat content, milk production, age structure, slaughtered animal population, and other relevant factors^2,18,33^. Emissions were calculated based on the 2019 refinement of the 2006 IPCC Guidelines for National Greenhouse Gas Inventories and referred to the guidelines published by China’s official authorities^32,34^. The inventory built in this study was validated via a comparison to previous studies (including results obtained with both bottom-up and top-down methods) and international datasets, and Monte Carlo simulation analysis was adopted in uncertainty analysis^35,36^. All related data description and data sources can be found in references and Supplementary Information (Table S9).

## Methods

Livestock methane emissions include emissions originating from enteric fermentation and manure management^37,38^. In enteric fermentation, the rumen microbiome of ruminant animals is responsible for methane production through the digestion of plant feed^37^. Methane emissions from manure management is caused by the anaerobic digestion conditions and is closely related to manure management practices^39^. Ruminant animals are the main contributors to livestock methane emissions, while nonruminant animals such as swine can also emit methane, especially in the manure management process^40^. There are currently two main approaches for estimating methane emissions, namely, top-down and bottom-up methods^41^. The top-down method usually involves satellite observation technology, while the bottom-up method is usually based on statistical data^42^. In this study, to construct a livestock methane emission inventory with a specific emission structure at the city level, the bottom-up method was adopted.

To comprehensively evaluate livestock methane emissions, ruminant animals (dairy cattle, nondairy cattle, buffalo, sheep, goats and camels) and nonruminant animals (swine, horses, donkeys, mules, poultry and rabbits) in each city of China were included in this study. In regard to enteric fermentation, key parameters, including live body weight, milk production and breeding system, were considered. Regarding species with small emission contributions, such as horses and donkeys, EFs were obtained based on the Guidelines for Provincial Greenhouse Gas Inventories (Trial) released by the National Development and Reform Commission (NDRC) of China^43^. In manure management, manure management practices, annual temperature and manure excretion volume were considered to improve the accuracy of our inventory. The detailed estimation methods and key parameters of the different livestock categories are provided in Table 1.

**Table 1:** Estimation methods and parameter descriptions of the different livestock categories: e denotes enteric fermentation, and m denotes manure management.

Categories	Classification	Methodological basis	Key parameters in EF determination (e)	Key parameters in EF determination (m)
Dairy cattle	Mature female/Young/Others	Tier 2	Milk production	Body weight/Manure management/Temperature
Non-dairy cattle	Mature female/Young/Others	Tier 2	Body weight/Breeding system/Productive performance	
Buffalo	Mature female/Young/Others	Tier 2		
Sheep	Mature female/Others	Tier 2	Body weight/ Productive performance /Breeding system	
Goat	Mature female/Others	Tier 2		
Swine	No classification	Tier 2	None	
Horse/Donkey/Mule, etc.	No classification	Tier 1	None	None

**Fig. 1:**
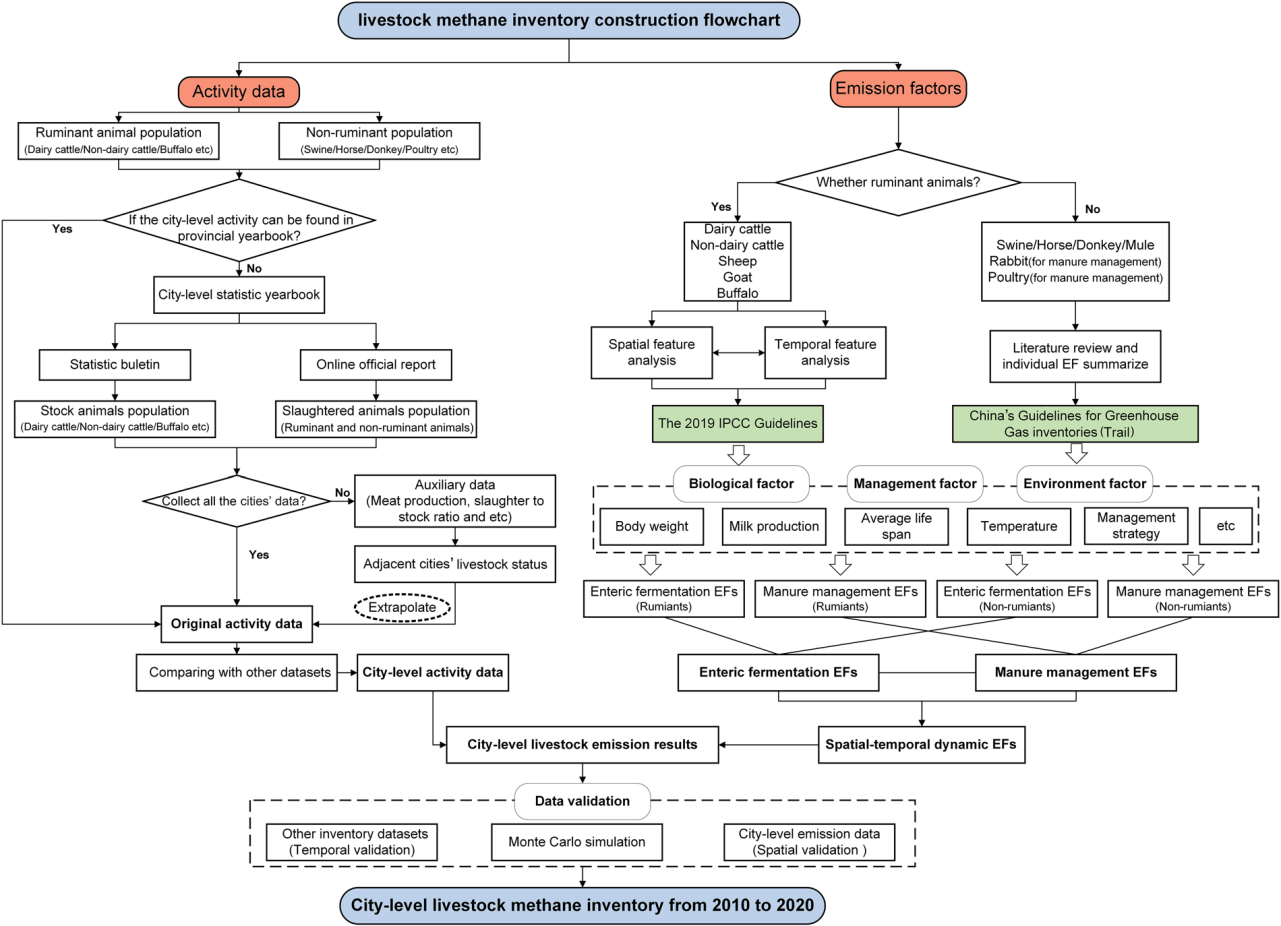
Flowchart of long-term city-level livestock methane emission estimation in this study.

A flowchart of long-term city-level livestock methane inventory construction is shown in Fig. 1. The first step was to collect city-level livestock activity data for each year, including stock and slaughter population data, for all prefecture-level cites. The second step involved building enteric fermentation EF data based on region-specific livestock parameters. The third step entailed determining the regional manure management strategy ratio and regional annual temperature and evaluating the volatile solid manure excreted for calculating manure management EFs of all livestock species. Finally, three validation strategies were adopted to assess the robustness of our inventory.

### City-level activity data collection and processing

We collected city-level livestock breeding data from various sources, including provincial statistical yearbooks, city statistical yearbooks, city statistical bulletins, online official reports, and municipal government records. Notably, publicly available resources such as the China Dairy Yearbook, China Animal Husbandry and Veterinary Yearbook, and China Agriculture Yearbook were considered^44–46^. Online official reports and local statistical yearbooks are released annually on the official government homepage and the Bureau of Statistics website for each city (e.g., https://www.km.gov.cn/c/2021-06-22/3985015.shtml). In cases where it is challenging to obtain direct data for cities with lower development levels, such as Baicheng, Shuangyashan, and Meishan, the necessary information can be acquired by establishing communication with the staff of the Bureau of Statistics through official websites, official email, and official disclosure. Through this approach, activity data can be acquired by anyone for reasonable applications. For a small number of cities (less than 5% of the total sample) with no livestock breeding records, we used the meat production of certain livestock categories and combined the slaughter rate in adjacent cities, animal live body weight, and carcass weight to extrapolate the stock or slaughtered animal population. The slaughter rate remains relatively stable within a city, so priority must be given to the slaughter rate of the city to replace any missing data. Finally, city-level activity data were collected, and the overall trend was validated via a comparison with provincial livestock data. In some provinces, the provincial and prefecture-level city data exhibit different statistical qualities, which may lead to inconsistency. Therefore, the purpose of validation was to ensure the overall data trend, and uncertainty was considered in uncertainty analysis. The uncertainty in the activity data is examined in the Data Validation section of this study. In summary, livestock activity data for 12 categories covering 347 cities in China were collected and prepared.

### Enteric fermentation-related methane emissions

In livestock breeding, the unique phycological processes in the rumen can result in the emission of large quantities of methane^37^. To calculate the EFs of enteric fermentation for the various ruminant animals, the gross energy intake of animals and the methane conversion factor (MCF) must be evaluated^2,34^. The gross energy intake basically includes the energy available for maintenance and growth, as expressed in Eq. 1.1. The gross energy (*GE*) can be divided into several aspects, including energy for maintenance (*NE*_*m*_), energy for animal activity (*NE*_a_), energy for lactation (*NE*_*l*_), energy for work (*NE*_*work*_), energy for maintaining pregnancy (*NE*_*p*_), energy for growth (*NE*_*g*_), and energy for producing wool (*NE*_*wool*_). Here, *EF*_*i, j*_ is methane emission factor for animal species *i* during enteric fermentation in *j* year, expressed in kg methane head^−1^ year^−1^. *Y*_*m*_ is the conversion factor, which indicates that a proportion of the gross energy is converted into methane, and 55.65 (MJ kg^−1^) is the energy content in methane^2,19,32^. Notably, the energy available for maintaining pregnancy and lactation is limited to mature females that can give birth, and growth is limited to young animals^32^. Additionally, the energy available for work was not included in the dataset, considering that draft animals in China are not sufficiently counted in the statistical yearbook, and they were increasingly replaced with farm machines in the 21^st^ century, especially in recent decades^47^. The detailed calculation process can be found in the Supplementary Information (Text S1). To produce more specific EFs of ruminant animals for methane emission estimation, *GE*_ij_ must be precisely accounted for by Eq. 1.2 according to the IPCC Tier 2 methodology.

1.1 $$E{F}_{i,j}=\frac{GE\times \left(\frac{{Y}_{{\rm{m}}}}{{\rm{100}}}\right)\times {\rm{365}}}{{\rm{55}}{\rm{.65}}}$$

1.2 $$GE=\frac{\left(\frac{N{E}_{m}+N{E}_{{\rm{a}}}+N{E}_{l}+N{E}_{work}+N{E}_{p}}{REM}\right)+\left(\frac{N{E}_{g}+N{E}_{wool}}{REG}\right)}{DE}$$

where *REM* is the proportion of the net energy available for maintenance in the digestible energy, *REG* is the ratio of the total energy available for growth to the digestible energy, and *DE* denotes the digestibility of feed reflected as the ratio of the digestible energy to the gross energy, as collected from the literature^18,48^. The detailed calculation procedure is described in the Supplementary Information (Text S1), which includes calculation methods for the different energy sources of gross energy.

Animal body weight, including energy for direct or indirect maintenance, activity and growth, is the most crucial parameter in gross energy calculation^11,18^. The temporal variation in the animal weight distribution can be acquired from the China Agricultural Products Cost‒benefit Information Compilation (CAPCIC) dataset^49,50^. For cities with no records for a specific year, the average change rate of the animal body weight was measured and combined with the average body weight in other years to obtain the live body weight. Here, we assumed that the body weight of the animals would remain stable and that there would be no abrupt changes. Regarding dairy cattle, the body weight was not recorded in the official documents. Instead, milk production and Eq. 1.3 were adopted for calculating the EF of dairy cattle based on the daily production in different regions and years^18^; these values were acquired from the China Dairy Yearbook^46^.

1.3 $$E{F}_{D{C}_{{\rm{i}}},j}=30.8\times productio{n}_{mil{k}_{i,j}}^{0.2}-53.6$$

where $E{F}_{D{C}_{i,j}}$ is the EF for dairy cattle in region *i* and year *j*, and $productio{n}_{mil{k}_{i,j}}$ denotes the milk production per head in region *i* and year *j*.

In addition, due to the differences in the body weight and productivity among different age groups, it is necessary to apply a classification system to ensure precision^51^. Here, when estimating methane emissions of ruminant animals, cattle were classified into mature male animals, young animals and others, and sheep/goats were divided into manure females and others. The EFs for nonruminant animals were obtained based on the Guidelines for Provincial Greenhouse Gas Inventories (Trial), which account for China’s actual situation^43^, because of the relatively low contribution to methane emissions and the poor availability of data for nonruminant animals.

### Manure management-related methane emissions

The livestock manure process can result in methane emission in an anaerobic environment, and the key factors are the volume of manure excreted and the manure management system used^52,53^. In an anaerobic environment, especially when liquid manure is stored, more methane is emitted^32^. Ignoring the difference between manure management systems and using default information for inventory construction could lead to high uncertainty in the emission results. Here, based on the 2019 refinement to IPCC guidelines, manure management practices, regional temperature conditions and livestock manure excoriation were considered for obtaining more specific EFs in the manure management process, as follows:

2.1 $${E}_{manure}=\sum _{T,S}{N}_{T}\times V{S}_{T}\times Managemen{t}_{T,S}\times E{F}_{T,S}$$

2.2 $$V{S}_{T}=V{S}_{rat{e}_{T}}\times \frac{A{M}_{T}}{1000}\times 365$$

2.3 $$E{F}_{T,S}=365\times V{S}_{T}\times \left({B}_{0(T)}\times 0.067\times \sum _{S,k}\frac{MC{F}_{S,k}}{100}\times Managemen{t}_{T,S}\right)$$

where, *E*_*manure*_ denotes the methane emissions from manure management of all livestock categories (g methane year^−1^), *N*_*T*_ is the animal population of category *T* in manure management system *S*, *VS*_*T*_ is the volatile solid excreted under category *T* in a given year, $Managemen{t}_{T,S}$ is the fraction of manure managed in system *S* of livestock category *T*. Here, the value of $Managemen{t}_{T,S}$ was obtained from the People’s Republic of China National Greenhouse Gas Inventory^51^. $E{F}_{T,S}$ is the emission factor of manure management of category *T* in system *S*, $V{S}_{rat{e}_{T}}$ is the manure excretion rate of livestock category *T* in units of 1,000 kg animal mass^−1^ day^−1^. Here, the value of $V{S}_{rat{e}_{T}}$ was obtained from the 2019 IPCC guidelines^32^. *AM*_*T*_ is the animal mass weight (kg), which was replaced with regional information to reduce the uncertainty in the constructed inventory. Detailed descriptions of dairy cattle, nondairy cattle, sheep/goats and swine are provided in Table S1 to Table S4. In regard to the EFs of manure management, ${B}_{0(T)}$ and $MC{F}_{S,k}$ denote the maximum methane emission capacity of manure of livestock category *T* and the methane conversion factor of manure management system *S* in climate region *k*, respectively. ${B}_{0(T)}$ is associated with animal manure and varies with the livestock category, but the latter depends on the manure management system, which reflects the degree of anaerobic conditions. In addition, methane emissions increase with increasing temperature^32^. Considering that China hosts a large animal breeding population and multiple climate regions, temperature can impact the methane conversion efficiency in manure management. Therefore, the regional temperature was considered in determining the MCF, and detailed information is provided in Table S5. The average temperature of each city was collected from the provincial statistical yearbooks, which are available on the official website of each province.

### Livestock methane emissions

Livestock methane emissions can be calculated as follows:

3.1 $${E}_{total,i}={E}_{e,i}+{E}_{m,i}$$

3.2 $${E}_{e,i}=\sum _{T,\beta =0,1}E{F}_{e,i}\times {N}_{T,i}\times AL{S}_{T,\beta }/12$$

3.3 $${E}_{m,i}=\sum _{T,\beta =0,1}E{F}_{m,i}\times {N}_{T,i}\times AL{S}_{T,\beta }/12$$

where ${E}_{total,i}$ denotes the total livestock methane emissions of all livestock categories in year *i*, and ${E}_{e,i}$ and ${E}_{m,i}$ denote the methane emissions resulting from enteric fermentation and manure management, respectively, and ${N}_{T,i}$ is the population of livestock category *T* in year *i*. The average lifespan ($AL{S}_{T,\beta }$) is the number of months that animals of livestock category *T* emit methane within a calendar year. This value also differs between stock and slaughtered animals. In regard to the stock population, *β* is 1, and a value of 0 is assigned to the slaughtered population. The livestock methane inventory must also consider the slaughtered animal population^18,19^ because of the high animal-derived food demand in China and the rapidly increasing slaughtered population. The slaughtered animal population emitted methane before death in the same year, although this amount is smaller than that of the stock population. Considering $AL{S}_{T,\beta }$ could also reduce the uncertainty in the results because the different livestock categories may exhibit different durations throughout the year, such as 12 months for stocked cattle and 5.6 months for stocked sheep. Regarding the slaughtered population, we assumed that it was evenly distributed in each year, and $AL{S}_{T,\beta }$ is smaller than that of the stock population, which was obtained from previous studies^18,19^. $AL{S}_{T,\beta }$ of the different animals is provided in the Supplementary Information (Table S6).

## Data Records

Our dataset contains city-level methane emissions from 2010 to 2020 (3817 data records), while provincial and national data were also recorded in the dataset. The dataset is made public under Figshare^54^. The enteric fermentation and manure management methane emissions of each city were also recorded in the dataset (7634 data records). Additionally, the contributions of each livestock category from 2010 to 2020 were provided in our dataset, and all the data were compiled in the XLSX file format. The following data are included:

1. A total of 347 cities (including autonomous regions) with 3817 livestock methane emission records (2010–2020) [file “China city-level livestock methane emissions, 2010 to 2020”];
2. A total of 347 cities (including autonomous regions) with 3817 records of livestock methane emissions resulting from enteric fermentation [file “China city-level livestock methane emissions from enteric fermentation, 2010 to 2020”];
3. A total of 347 cities (including autonomous regions) with 3817 records of livestock methane emissions from manure management [file “China city-level livestock methane emissions from manure management, 2010 to 2020”];
4. A total of 347 cities (including autonomous regions) with 7634 records of livestock methane emissions of ruminant and nonruminant animals [file “China city-level methane emissions from ruminant and nonruminant animals, 2010–2020”];
5. National and provincial livestock methane emissions and the structure of enteric fermentation and manure management emissions [file “China national and provincial livestock methane emission inventory, 2010–2020”];
6. Emissions of each livestock species from 2010 to 2020 [file “Methane emissions of each livestock category, 2010–2020”].

This livestock methane inventory provides city-level methane emission data for 12 livestock categories and 347 prefecture-level cities (autonomous regions) in China from 2010 to 2020. This dataset can be used for further analysis of livestock emissions, thereby focusing on food consumption, climate change, agricultural development, dietary transition, etc. The livestock methane emissions in more than 300 prefecture-level cities in China exhibited a significant change in the spatial distribution pattern in 2010, 2015 and 2020. Detailed spatial distribution results for the city-level livestock methane emissions in China are provided in the file “China city-level livestock methane emissions, 2010 to 2020”. As one of the features of this dataset (relative to previous data), the spatial-temporal distribution characteristics of city-level livestock methane emissions can be captured as a long time series. Additionally, methane emissions resulting from enteric fermentation and manure management at the city level can be found in this dataset for further analysis. Cattle, especially nondairy cattle, constitute the largest methane emitters in the enteric fermentation process, and swine contribute more than 50% to the total emissions in the manure management process.

## Technical Validation

### Uncertainty analysis

**Fig. 2:**
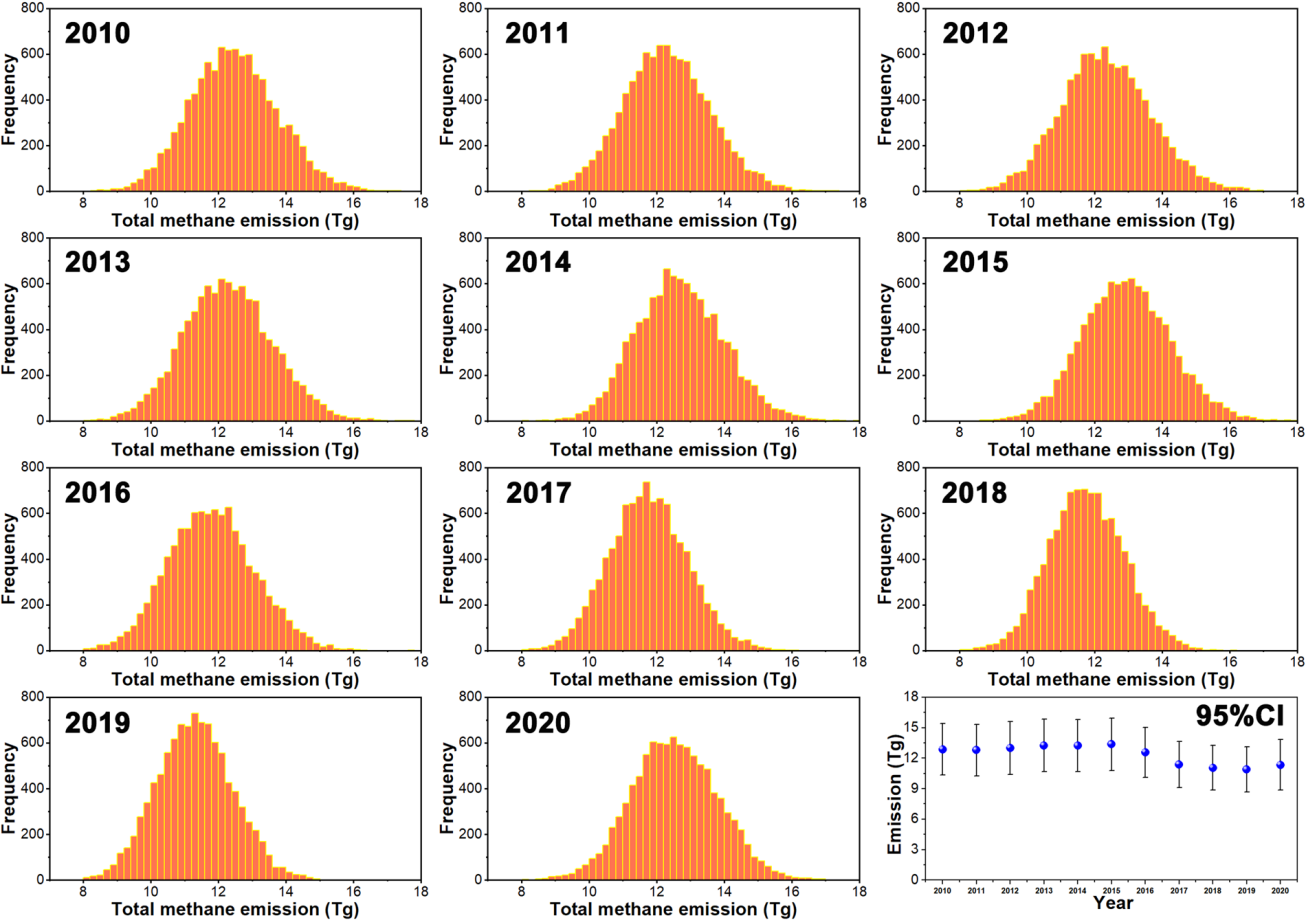
Distribution of the emissions in each year obtained via Monte Carlo simulation (2010 to 2020). The last error distribution (blue points) indicates the estimated emission and the standard deviation of 10,000 runs.

The uncertainty in the activity data depends on the data collection methods and scale^11^. For instance, the population of certain animals may vary at the city, country and international levels due to differences in the quantity of statistics and statistical quality^55^. The uncertainty in the EFs originates from the estimation process, which involves numerous parameters. The Monte Carlo method is a numerical computing methodology that involves statistical simulation and random sampling to address uncertainty problems and is widely used in uncertainty analysis studies of emission inventories. In general, first, the 95% CI of the probability density function (PDF) of each variable must be calculated, and random values are then selected from the PDF to calculate emission values. Monte Carlo analysis usually entails repeating the calculation a statistically significant number of times ranging from 100 to 10,000. This process yields the PDF of each variable, including the activity data and EFs for the different animal categories^16,56,57^. The distribution of the EFs in this study is assumed to be a normal distribution according to the 2006 IPCC Guidelines for National Greenhouse Gas Inventories^34^. Regarding the activity data, we assumed that they followed a uniform distribution according to previous studies^11,33^. The activity data in our study were compared to different activity data datasets to account for any potential uncertainty, including data from China Statistical Yearbook and international statistical datasets (FAOSTAT). Xu *et al*.^11,33^ used the Monte Carlo simulation method to validate the uncertainty in their livestock methane or nitrous oxide inventory, and the uncertainty in both the EF values and activity data was considered. According to Xu, the uncertainty in the activity data was analyzed via a comparison to other datasets. In the analysis, the uncertainty stemmed from the activity data, and EFs were separately estimated. The coefficient of variation (CV) of the activity data can be defined as the absolute value of the average difference among the three activity population datasets normalized by the mean of these datasets, and we assumed a uniform distribution for the activity data according to previous studies (Table S7)^11,33,58^. The distribution of EFs was assumed to be a normal distribution, and the CVs of the EFs of each livestock category, expressed as the standard deviation relative to the mean, were collected from the literature (Table S8)^33,59,60^. Then, 10,000 independent simulations were conducted to estimate the range of methane emissions. Finally, the 95% confidence interval (CI) was used to characterize the uncertainty in our inventory. The simulation results are shown in Fig. 2, and the maximum uncertainty across all years varies between −22% and 18% (more detailed information can be found in Table S10).

### Comparisons with other datasets

**Fig. 3:**
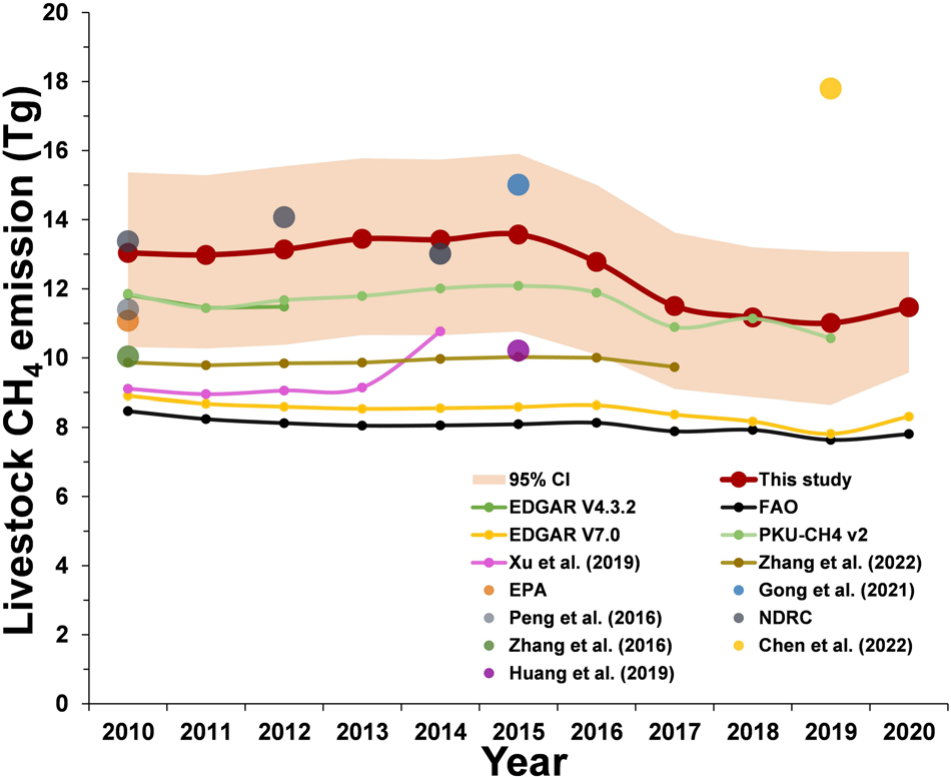
Uncertainty and comparison with other datasets or research results at the national level. The points indicate single-year results, and the lines indicate time-series emission results. The red shadow indicates the 95% confidence interval of the Monte Carlo simulation results involving 10,000 runs.

To ensure the reliability of our inventory, it is necessary to compare our results with those of previous datasets or studies, especially by providing a comprehensive comparison of both bottom-up and top-down results^61–65^. Two comparison pathways were considered here because few city-level methane emission inventories are available, especially long-term series datasets. First, we compared our emission results at the national level with existing official datasets and high-quality published studies. Figure 3 shows the verification results of national emission dynamics. Our emission variation trend is similar to that in most previous studies, with a stable trend beginning in 2010, followed by a downward trend after 2015 and an increasing trend in approximately 2020. The FAO- and EDGAR v7.0-based results differ the most from our estimates, which are 35% (29%–40%) and 32% (27–37%) lower, respectively. The main reason is that constant EFs were used in their estimation process, which are replaced with region-specific EFs in our approach. Another important reason is that the activity data widely differ between the FAO data and Chinese statistics (EDGAR also uses FAO activity data), which may lead to underestimation of the livestock population in China^2,18^. It has been indicated that constant EFs may cause underestimation of the change in livestock emissions, which may lead to higher Tier 2 method results than Tier 1 results for China^18,19^. EDGAR v4.3.2 considers the milk yield of dairy cattle and animal weight, and the results were 11% (9%–13%) lower than our estimates. Therefore, these results are more similar to our estimates than those of EDGAR v7.0. Our estimates are more consistent with those of the PKU-CH_4_ dataset, especially after 2016. The difference between our estimates and the PKU-CH_4_ concentration data remains less than 12%, or even less than 5%, over the last five years (0.3%–12%). The consistency between our estimates and the PKU-CH_4_ concentration data can be mainly attributed to the use of region-specific EFs; moreover, the average lifespan and slaughtered animal population were considered in their estimation process. The emissions of the NDRC (published by the official department of China) are also consistent with our estimates, with an average difference of 2% (−3%–7%), thereby adopting more relevant activity data and county-specific EFs for estimation. Regarding the results of Xu *et al*.^11^, the emission data are significantly lower than our estimates and other results by 29% (20%–32%), mainly due to the adoption of county-level activity data obtained, which may have higher uncertainty. Regarding the estimates of the top-down method, our results are between those of Zhang *et al*.^62^ and Chen *et al*.^61^. Nevertheless, the livestock sector’s methane estimation has the larger uncertainty based on top-down method than other sectors^61^. In regard to other one-year emission records, the main reason for the differences is the use of constant EFs by them, referred to as the IPCC Tier 1 method, results in emission underestimation.

**Fig. 4:**
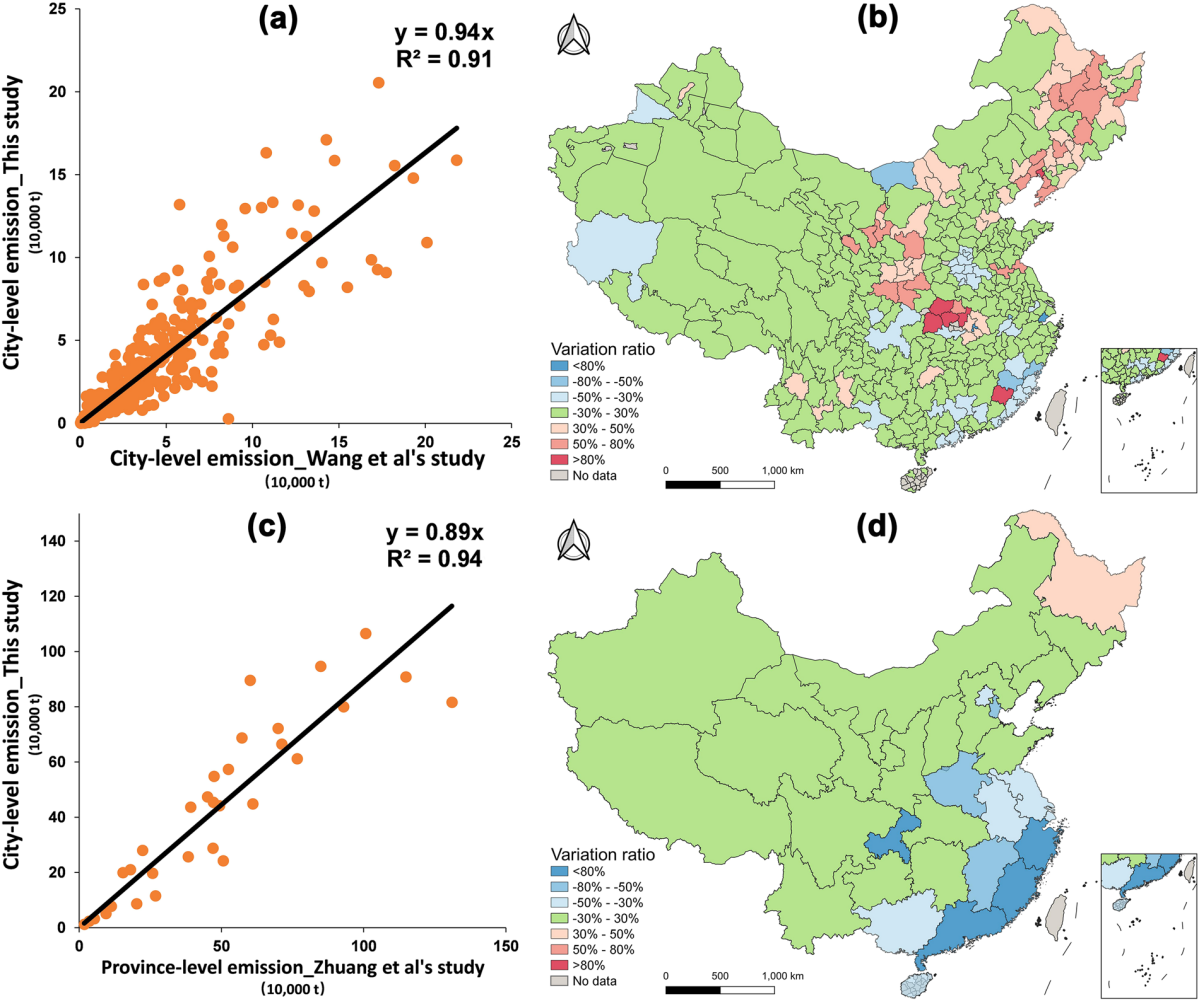
Comparison to existing city-level inventories for 2015. (**a**). Fitting results between our results and those of Wang *et al*. (**b**). Spatial validation between our results and those of Wang *et al*. (**c**). Fitting results between our results and those of Zhuang *et al*. (**d**). Spatial validation between our results and those of Zhuang *et al*.

Second, we compared our spatial emission characteristics at the city level with the only available study on detailed city-level livestock methane emissions in 2015 by Wang *et al*.^66^. The authors used default EFs to estimate livestock methane emissions in China. Although this study did not fully adopt the Tier 2 method and considered only one year of data, with the same spatial scale as that of our study (which has not been employed in other studies) and a relatively stable spatial distribution pattern (relative stability of hot and cold spots of emissions), we used this dataset for spatial validation. The spatial distribution pattern of livestock methane emissions in our dataset was subsequently compared with the results of Wang *et al*.^66^, as shown in Fig. 4, revealing a significant linear trend (R^2^ = 0.91). Therefore, our results for 2015 are highly consistent with the city-level results of Wang *et al*. in terms of emission values, with differences less than −30% to 30% in most cities. However, spatial variation could still be observed, particularly in cities in Northeast China. The main reasons for this finding are the consideration of more detailed biological issues, manure management strategies, livestock breeding structure and slaughtered populations in our studies, which are not considered in the inventory of Wang *et al*. Additionally, some outliers could be explained by the updated activity data used in our inventory, especially for the cities of Yichang, Xiangyang, Jinmen and Longyan, etc. To further validate our results against a province-level emission inventory, we compared our results to those of Zhuang *et al*.^17^, who evaluated livestock methane emissions at the province level. In their study, mean EFs of enteric fermentation from other studies were adopted, and region-specific EFs were considered the calculation of manure management emissions. Figure 4(c,b) shows that our results exhibit a significant linear trend (R^2^ = 0.94), and most provinces demonstrate differences between −30% and 30%.

### Limitations

In summary, our dataset provides up-to-date and finer-spatial resolution livestock methane emissions at the city level, which could bridge the data gap and serve as a reference for related studies. However, there are still two main limitations that must be noted and addressed in future work. First, although our inventory includes most of the livestock categories raised in China, some ruminant animals, such as deer and alpaca, are missing in the inventory due to the lack of statistical animal population data. Even though the population of these livestock categories is small, they should be included when data become available. Ignoring minority livestock species may result in underestimation of the emissions. Second, to conform with China’s livestock methane emission conditions, we consider biological, management, and climate conditions. Nevertheless, different farms or breeding plants may exhibit unique emission factors due to variations in breeding technology and location. The differentiated breeding practices may cause changes in EFs. These factors can be monitored and measured using various techniques, such as eddy covariance techniques, tracer methods, and high-resolution unmanned aerial vehicles, to achieve a more precise estimation^67–69^. A more detailed estimation based on an observation system or instruments is a potential way to further reduce the estimation uncertainty. Additionally, uncertainty is inevitable in statistical data due to the statistical quality. The uncertainty in the statistical data and the process of replacing missing data can be improved in the future.

### Supplementary information

Supplementary Information

## Data Availability

The datasets are available in the form of XLSX files. No specific code was used to construct the datasets.
