# Supplementary material for: City-level livestock methane emissions in China from 2010 to 2020
Source: Sci Data. 2024 Feb 28;11:251. doi: 10.1038/s41597-024-03072-y (PMC10902353; doi:10.1038/s41597-024-03072-y)
Supplement: Supplementary file 1 — Supplementary Information [file 41597_2024_3072_MOESM1_ESM.pdf]

**Supplementary Information of**

# City-level livestock methane emissions in China from 2010 to 2020

Mingxi Du*[1], Xiang Kang[1], Qiuyu Liu[1], Haifeng Du[1], Jianjun Zhang[2], Yulong Yin[3], Zhenling Cui[3]

1. School of Public Policy and Administration, Xi'an Jiaotong University, Xi'an, 710049 China
2. School of Land Science and Technology, China University of Geosciences, Beijing 100083, China
3. College of Resources and Environmental Sciences, National Academy of Agriculture Green Development, Key Laboratory of Plant-Soil Interactions, Ministry of Education, China Agricultural University, Beijing 100193, China

*Corresponding author: M.D. (dumingxi28@xjtu.edu.cn)

**Contents of this file**

**Text S1**

**Table S1.** Swine manure management system ratio in different regions.

**Table S2.** Dairy cattle manure management system ratio in different regions.

**Table S3.** Non-dairy cattle manure management system ratio in different regions.

**Table S4.** Sheep/Goat manure management system ratio in different regions.

**Table S5.** Methane conversion factors in different mean annual temperature (%).

**Table S6.** Average life span (ALS) of different livestock animals (month).

**Table S7.** Coefficients of variation of activity data among different dataset (city level, country level and international level).

**Table S8.** Coefficients of variation of EFs of different livestock categories.

**Table S9.** Data sources of the main data in this study.

**Table S10.** The uncertainty interval of the final emission results.

## Text S1

For comprehensively estimating the gross energy ( $GE_{ij}$ ) of large ruminants, including non-dairy cattle, dairy cattle, buffalo, sheep and goats. Energy for maintenance ( $NE_m$ ), energy for animal activity ( $NE_a$ ), energy for lactation ( $NE_l$ ), energy for pregnancy ( $NE_p$ ), energy for growth ( $NE_g$ ) and energy for producing wool ( $NE_{wool}$ ) were all considered in the determination of $GE_{ij}$ according to IPCC 2019 and previous studies[1,2]. It is noted that the energy for pregnancy and lactation is just for mature females who give birth, and the growth is only for young animals.

The energy for maintenance ( $NE_m$ ) is the energy animals used to maintain their life, and is the function of body weight as follows.

$$NE_m = Cf_i \times weight_{i,j}^{0.75} \qquad \text{S1}$$

where $Cf_i$ is the coefficient varies over livestock categories math to the animal $i$ , and the value of which refers to IPCC 2019 inventory guidelines[1], and the $weight$ is the body weight of the animal $i$ in the year $j$ .

The energy for animal activity ( $NE_a$ ) is net energy for activity, including obtaining food and etc. the value of $NE_a$ is defined as a part of $NE_a$ as follows, S2 is for cattle and buffalo, and S3 is for sheep and goats.

$$NE_a = C \times NE_m \qquad \text{S2}$$

$$NE_a = C \times weight_{i.j} \qquad \text{S3}$$

where $C$ is the coefficient determined by the feeding situation, and according to previous studies and the current situation of China's livestock breeding factor, Qinghai, Xinjiang, Tibet, and Inner Mongolia were mainly in the grazing system. The corresponding $C$ value can be found in IPCC 2019 inventory guidelines[1].

The energy for lactation ( $NE_l$ ) is only for mature female animals as follows,

$$NE_L = milk \times (1.47 + 0.4 \times fat) \qquad \text{S4}$$

where $milk$ is the amount of milk production and the $fat$ is the content of milk which all can be found in China diary yearbook[3].

The energy for pregnancy ( $NE_p$ ) is also only for mature female animals as follows, the S5 is for non-dairy cattle and buffalo, S7 is for sheep and goats.

$$NE_g = 22.02 \times \frac{weight_{i,j}}{c \times weight_m} \times WG^{1.097} \qquad \text{S5}$$

$$WG = \frac{weight_m - weight_y}{age_m - age_y} \qquad \text{S6}$$

$$NE_g = \frac{WG \times (a + 0.5 \times b \times (BW_i + BW_f))}{365} \qquad \text{S7}$$

where $c$ is the coefficient of 0.8, 1.0 and 1.2 for females, castrates and bulls, respectively, due to the data available, the mean value was adopted in this study. The $weight_m$ is the weight of the mature body of the certain animal, $WG$ is the wright gain of young animals which can be measured as S6, the $weight_y$ is the body weight of the young animal, the $age_m$ and $age_y$ is the age of mature and young animals, respectively[2]. In S7, $BW_i$ is the bodyweight of animals at weaning, and $BW_f$ is at 1-year old with the unit of kg, due to lack of detailed information about sheep and goats, according to Xu's study, the value of $WG$ was set as 0.15 kg, and the body weight at weaning and 1-year old was

assumed is same to the weight of young and mature animals. Additionally, the value of a and b in S7 can be found in IPCC 2019 inventory guidelines[1].

The energy for producing wool ( $NE_{wool}$ ) is the net energy required to produce wool in a year for sheep and goats, and the equation is as follows,

$$NE_{\text{wool}} = \frac{E_{wool} \times P_{wool}}{365} \quad \text{S8}$$

where $E_{wool}$ is the energy value for sheep/goats to produce each kg of wool, which is 24 MJ $kg^{-1}$, and $P_{wool}$ is the wool production in a year per head (kg $year^{-1}$)[1]. Due to the lack of specific wool production data, here we use the provincial level data through estimation combining the total wool production and the population of sheep/goats.

| ID | city | Solid storage | Dry lot | Liquid storage | Daily spread | Anaerobic digestion | Combustion | others |
|---|---|---|---|---|---|---|---|---|
| 1 | Zunyi | 20% | 18% | 13% | 0% | 19% | 19% | 30% |
| 2 | Zigong | 20% | 18% | 13% | 0% | 19% | 19% | 30% |
| 3 | Zibo | 15% | 45% | 9% | 0% | 20% | 20% | 11% |
| 4 | Ziyang | 20% | 18% | 13% | 0% | 19% | 19% | 30% |
| 5 | Zhumadian | 15% | 45% | 9% | 0% | 20% | 20% | 11% |
| 6 | Zhuzhou | 20% | 18% | 13% | 0% | 19% | 19% | 30% |
| 7 | Zhuhai | 20% | 18% | 13% | 0% | 19% | 19% | 30% |
| 8 | Zhoukou | 15% | 45% | 9% | 0% | 20% | 20% | 11% |
| 9 | Zhoushan | 14% | 31% | 8% | 0% | 25% | 25% | 22% |
| 10 | Chongqing | 20% | 18% | 13% | 0% | 19% | 19% | 30% |
| 11 | Zhongwei | 30% | 30% | 20% | 0% | 20% | 20% | 0% |
| 12 | Zhongshan | 20% | 18% | 13% | 0% | 19% | 19% | 30% |
| 13 | Zhengzhou | 15% | 45% | 9% | 0% | 20% | 20% | 11% |
| 14 | Zhenjiang | 14% | 31% | 8% | 0% | 25% | 25% | 22% |
| 15 | Zhaoqing | 20% | 18% | 13% | 0% | 19% | 19% | 30% |
| 16 | Zhaotong | 20% | 18% | 13% | 0% | 19% | 19% | 30% |
| 17 | Changzhi | 15% | 45% | 9% | 0% | 20% | 20% | 11% |
| 18 | Changsha | 20% | 18% | 13% | 0% | 19% | 19% | 30% |
| 19 | Changchun | 11% | 50% | 28% | 0% | 10% | 10% | 1% |
| 20 | Zhangzhou | 14% | 31% | 8% | 0% | 25% | 25% | 22% |
| 21 | Zhangye | 30% | 30% | 20% | 0% | 20% | 20% | 0% |
| 22 | Zhangjiakou | 15% | 45% | 9% | 0% | 20% | 20% | 11% |
| 23 | Zhangjiajie | 20% | 18% | 13% | 0% | 19% | 19% | 30% |
| 24 | Zhanjiang | 20% | 18% | 13% | 0% | 19% | 19% | 30% |
| 25 | Zaozhuang | 15% | 45% | 9% | 0% | 20% | 20% | 11% |
| 26 | Yuncheng | 15% | 45% | 9% | 0% | 20% | 20% | 11% |
| 27 | Yunfu | 20% | 18% | 13% | 0% | 19% | 19% | 30% |
| 28 | Yueyang | 20% | 18% | 13% | 0% | 19% | 19% | 30% |
| 29 | Yuxi | 20% | 18% | 13% | 0% | 19% | 19% | 30% |
| 30 | Yushu | 30% | 30% | 20% | 0% | 20% | 20% | 0% |

| | | | | | | | | |
|---|---|---|---|---|---|---|---|---|
| 31 | Yulin | 20% | 18% | 13% | 0% | 19% | 19% | 30% |
| 32 | Yulin | 30% | 30% | 20% | 0% | 20% | 20% | 0% |
| 33 | Yongzhou | 20% | 18% | 13% | 0% | 19% | 19% | 30% |
| 34 | Yingkou | 11% | 50% | 28% | 0% | 10% | 10% | 1% |
| 35 | Yingtan | 20% | 18% | 13% | 0% | 19% | 19% | 30% |
| 36 | Yinchuan | 30% | 30% | 20% | 0% | 20% | 20% | 0% |
| 37 | Yiyang | 20% | 18% | 13% | 0% | 19% | 19% | 30% |
| 38 | Yichun | 20% | 18% | 13% | 0% | 19% | 19% | 30% |
| 39 | Yichang | 20% | 18% | 13% | 0% | 19% | 19% | 30% |
| 40 | Yibin | 20% | 18% | 13% | 0% | 19% | 19% | 30% |
| 41 | Ili Kazak | 30% | 30% | 20% | 0% | 20% | 20% | 0% |
| 42 | Yichun | 11% | 50% | 28% | 0% | 10% | 10% | 1% |
| 43 | Yangquan | 15% | 45% | 9% | 0% | 20% | 20% | 11% |
| 44 | Yangjiang | 20% | 18% | 13% | 0% | 19% | 19% | 30% |
| 45 | Yangzhou | 14% | 31% | 8% | 0% | 25% | 25% | 22% |
| 46 | Yancheng | 14% | 31% | 8% | 0% | 25% | 25% | 22% |
| 47 | Yanbian Korean | 11% | 50% | 28% | 0% | 10% | 10% | 1% |
| 48 | Yan‘an | 30% | 30% | 20% | 0% | 20% | 20% | 0% |
| 49 | Yantai | 15% | 45% | 9% | 0% | 20% | 20% | 11% |
| 50 | Ya'an | 20% | 18% | 13% | 0% | 19% | 19% | 30% |
| 51 | Xuancheng | 14% | 31% | 8% | 0% | 25% | 25% | 22% |
| 52 | Xuchang | 15% | 45% | 9% | 0% | 20% | 20% | 11% |
| 53 | Xuzhou | 14% | 31% | 8% | 0% | 25% | 25% | 22% |
| 54 | Xingan league | 15% | 45% | 9% | 0% | 20% | 20% | 11% |
| 55 | Xingtai | 15% | 45% | 9% | 0% | 20% | 20% | 11% |
| 56 | Xinyang | 15% | 45% | 9% | 0% | 20% | 20% | 11% |
| 57 | Xinyu | 20% | 18% | 13% | 0% | 19% | 19% | 30% |
| 58 | Xinxiang | 15% | 45% | 9% | 0% | 20% | 20% | 11% |
| 59 | Xinzhou | 15% | 45% | 9% | 0% | 20% | 20% | 11% |
| 60 | Xiaogan | 20% | 18% | 13% | 0% | 19% | 19% | 30% |
| 61 | Xiangyang | 20% | 18% | 13% | 0% | 19% | 19% | 30% |
| 62 | Xiangxi | 20% | 18% | 13% | 0% | 19% | 19% | 30% |
| 63 | Xiangtan | 20% | 18% | 13% | 0% | 19% | 19% | 30% |
| 64 | Xianyang | 30% | 30% | 20% | 0% | 20% | 20% | 0% |
| 65 | Xianning | 20% | 18% | 13% | 0% | 19% | 19% | 30% |
| 66 | Xiantao | 20% | 18% | 13% | 0% | 19% | 19% | 30% |
| 67 | Xilingol League | 15% | 45% | 9% | 0% | 20% | 20% | 11% |
| 68 | Xishuangbanna Dai | 20% | 18% | 13% | 0% | 19% | 19% | 30% |
| 69 | Xining | 30% | 30% | 20% | 0% | 20% | 20% | 0% |
| 70 | Xi'an | 30% | 30% | 20% | 0% | 20% | 20% | 0% |

| | | | | | | | | |
|---|---|---|---|---|---|---|---|---|
| 71 | Wuwei | 30% | 30% | 20% | 0% | 20% | 20% | 0% |
| 72 | Wuhan | 20% | 18% | 13% | 0% | 19% | 19% | 30% |
| 73 | Wuzhishan | 20% | 18% | 13% | 0% | 19% | 19% | 30% |
| 74 | Wuzhou | 20% | 18% | 13% | 0% | 19% | 19% | 30% |
| 75 | Wuzhong | 30% | 30% | 20% | 0% | 20% | 20% | 0% |
| 76 | Wuhu | 14% | 31% | 8% | 0% | 25% | 25% | 22% |
| 77 | Wuxi | 14% | 31% | 8% | 0% | 25% | 25% | 22% |
| 78 | Urumqi | 30% | 30% | 20% | 0% | 20% | 20% | 0% |
| 79 | Ulaanchabu | 15% | 45% | 9% | 0% | 20% | 20% | 11% |
| 80 | Wuhai | 15% | 45% | 9% | 0% | 20% | 20% | 11% |
| 81 | Wenshan | 20% | 18% | 13% | 0% | 19% | 19% | 30% |
| 82 | Wenchang | 20% | 18% | 13% | 0% | 19% | 19% | 30% |
| 83 | Wenzhou | 14% | 31% | 8% | 0% | 25% | 25% | 22% |
| 84 | Weinan | 30% | 30% | 20% | 0% | 20% | 20% | 0% |
| 85 | Weifang | 15% | 45% | 9% | 0% | 20% | 20% | 11% |
| 86 | Weihai | 15% | 45% | 9% | 0% | 20% | 20% | 11% |
| 87 | Wanning | 20% | 18% | 13% | 0% | 19% | 19% | 30% |
| 88 | Turpan | 30% | 30% | 20% | 0% | 20% | 20% | 0% |
| 89 | Tongren | 20% | 18% | 13% | 0% | 19% | 19% | 30% |
| 90 | Tongling | 14% | 31% | 8% | 0% | 25% | 25% | 22% |
| 91 | Tongchuan | 30% | 30% | 20% | 0% | 20% | 20% | 0% |
| 92 | Tongliao | 15% | 45% | 9% | 0% | 20% | 20% | 11% |
| 93 | Tonghua | 11% | 50% | 28% | 0% | 10% | 10% | 1% |
| 94 | Tieling | 11% | 50% | 28% | 0% | 10% | 10% | 1% |
| 95 | Tianshui | 30% | 30% | 20% | 0% | 20% | 20% | 0% |
| 96 | Tianmen | 20% | 18% | 13% | 0% | 19% | 19% | 30% |
| 97 | Tianjin | 15% | 45% | 9% | 0% | 20% | 20% | 11% |
| 98 | Tangshan | 15% | 45% | 9% | 0% | 20% | 20% | 11% |
| 99 | Taizhou | 14% | 31% | 8% | 0% | 25% | 25% | 22% |
| 100 | Tai'an | 15% | 45% | 9% | 0% | 20% | 20% | 11% |
| 101 | Taiyuan | 15% | 45% | 9% | 0% | 20% | 20% | 11% |
| 102 | Taizhou | 14% | 31% | 8% | 0% | 25% | 25% | 22% |
| 103 | Tacheng | 30% | 30% | 20% | 0% | 20% | 20% | 0% |
| 104 | Suining | 20% | 18% | 13% | 0% | 19% | 19% | 30% |
| 105 | Suizhou | 20% | 18% | 13% | 0% | 19% | 19% | 30% |
| 106 | Suihua | 11% | 50% | 28% | 0% | 10% | 10% | 1% |
| 107 | Suzhou | 14% | 31% | 8% | 0% | 25% | 25% | 22% |
| 108 | Suqian | 14% | 31% | 8% | 0% | 25% | 25% | 22% |
| 109 | Suzhou | 14% | 31% | 8% | 0% | 25% | 25% | 22% |
| 110 | Songyuan | 11% | 50% | 28% | 0% | 10% | 10% | 1% |
| 111 | Siping | 11% | 50% | 28% | 0% | 10% | 10% | 1% |
| 112 | Shuozhou | 15% | 45% | 9% | 0% | 20% | 20% | 11% |
| 113 | Shuangyashan | 11% | 50% | 28% | 0% | 10% | 10% | 1% |

| | | | | | | | | |
|---|---|---|---|---|---|---|---|---|
| 114 | Shizuishan | 30% | 30% | 20% | 0% | 20% | 20% | 0% |
| 115 | Shijiazhuang | 15% | 45% | 9% | 0% | 20% | 20% | 11% |
| 116 | Shiyan | 20% | 18% | 13% | 0% | 19% | 19% | 30% |
| 117 | Shenyang | 11% | 50% | 28% | 0% | 10% | 10% | 1% |
| 118 | Shennongjia | 20% | 18% | 13% | 0% | 19% | 19% | 30% |
| 119 | Shenzhen | 20% | 18% | 13% | 0% | 19% | 19% | 30% |
| 120 | Shaoxing | 14% | 31% | 8% | 0% | 25% | 25% | 22% |
| 121 | Shaoyang | 20% | 18% | 13% | 0% | 19% | 19% | 30% |
| 122 | Shaoguan | 20% | 18% | 13% | 0% | 19% | 19% | 30% |
| 123 | Shangrao | 20% | 18% | 13% | 0% | 19% | 19% | 30% |
| 124 | Shanghai | 14% | 31% | 8% | 0% | 25% | 25% | 22% |
| 125 | Shangqiu | 15% | 45% | 9% | 0% | 20% | 20% | 11% |
| 126 | Shangluo | 30% | 30% | 20% | 0% | 20% | 20% | 0% |
| 127 | Shanwei | 20% | 18% | 13% | 0% | 19% | 19% | 30% |
| 128 | Shantou | 20% | 18% | 13% | 0% | 19% | 19% | 30% |
| 129 | Shannan | 30% | 30% | 20% | 0% | 20% | 20% | 0% |
| 130 | Xiamen | 14% | 31% | 8% | 0% | 25% | 25% | 22% |
| 131 | Sanya | 20% | 18% | 13% | 0% | 19% | 19% | 30% |
| 132 | Sanming | 14% | 31% | 8% | 0% | 25% | 25% | 22% |
| 133 | Sanmenxia | 15% | 45% | 9% | 0% | 20% | 20% | 11% |
| 134 | Rizhao | 15% | 45% | 9% | 0% | 20% | 20% | 11% |
| 135 | Shigatse | 30% | 30% | 20% | 0% | 20% | 20% | 0% |
| 136 | Quanzhou | 14% | 31% | 8% | 0% | 25% | 25% | 22% |
| 137 | Quzhou | 14% | 31% | 8% | 0% | 25% | 25% | 22% |
| 138 | Qujing | 20% | 18% | 13% | 0% | 19% | 19% | 30% |
| 139 | Qionghai | 20% | 18% | 13% | 0% | 19% | 19% | 30% |
| 140 | Qingyang | 30% | 30% | 20% | 0% | 20% | 20% | 0% |
| 141 | Qingyuan | 20% | 18% | 13% | 0% | 19% | 19% | 30% |
| 142 | Qingdao | 15% | 45% | 9% | 0% | 20% | 20% | 11% |
| 143 | Qinhuangdao | 15% | 45% | 9% | 0% | 20% | 20% | 11% |
| 144 | Qinzhou | 20% | 18% | 13% | 0% | 19% | 19% | 30% |
| 145 | Qianxinan | 20% | 18% | 13% | 0% | 19% | 19% | 30% |
| 146 | Qiannan | 20% | 18% | 13% | 0% | 19% | 19% | 30% |
| 147 | Qiandongnan | 20% | 18% | 13% | 0% | 19% | 19% | 30% |
| 148 | Qianjiang | 20% | 18% | 13% | 0% | 19% | 19% | 30% |
| 149 | Qiqihar | 11% | 50% | 28% | 0% | 10% | 10% | 1% |
| 150 | Qitaihe | 11% | 50% | 28% | 0% | 10% | 10% | 1% |
| 151 | Pu'er | 20% | 18% | 13% | 0% | 19% | 19% | 30% |
| 152 | Puyang | 15% | 45% | 9% | 0% | 20% | 20% | 11% |
| 153 | Putian | 14% | 31% | 8% | 0% | 25% | 25% | 22% |
| 154 | Pingxiang | 20% | 18% | 13% | 0% | 19% | 19% | 30% |
| 155 | Pingliang | 30% | 30% | 20% | 0% | 20% | 20% | 0% |
| 156 | Pingdingshan | 15% | 45% | 9% | 0% | 20% | 20% | 11% |

| | | | | | | | | |
|---|---|---|---|---|---|---|---|---|
| 157 | Panjin | 11% | 50% | 28% | 0% | 10% | 10% | 1% |
| 158 | Panzhihua | 20% | 18% | 13% | 0% | 19% | 19% | 30% |
| 159 | Nujiang Lisu | 20% | 18% | 13% | 0% | 19% | 19% | 30% |
| 160 | Ningde | 14% | 31% | 8% | 0% | 25% | 25% | 22% |
| 161 | Ningbo | 14% | 31% | 8% | 0% | 25% | 25% | 22% |
| 162 | Neijiang | 20% | 18% | 13% | 0% | 19% | 19% | 30% |
| 163 | Nanyang | 15% | 45% | 9% | 0% | 20% | 20% | 11% |
| 164 | Nantong | 14% | 31% | 8% | 0% | 25% | 25% | 22% |
| 165 | Nanping | 14% | 31% | 8% | 0% | 25% | 25% | 22% |
| 166 | Nanning | 20% | 18% | 13% | 0% | 19% | 19% | 30% |
| 167 | Nanjing | 14% | 31% | 8% | 0% | 25% | 25% | 22% |
| 168 | Nnanchong | 20% | 18% | 13% | 0% | 19% | 19% | 30% |
| 169 | Nanchang | 20% | 18% | 13% | 0% | 19% | 19% | 30% |
| 170 | Nagchu | 30% | 30% | 20% | 0% | 20% | 20% | 0% |
| 171 | Mudanjiang | 11% | 50% | 28% | 0% | 10% | 10% | 1% |
| 172 | Mianyang | 20% | 18% | 13% | 0% | 19% | 19% | 30% |
| 173 | Meizhou | 20% | 18% | 13% | 0% | 19% | 19% | 30% |
| 174 | Meishan | 20% | 18% | 13% | 0% | 19% | 19% | 30% |
| 175 | Maoming | 20% | 18% | 13% | 0% | 19% | 19% | 30% |
| 176 | Ma'anshan | 14% | 31% | 8% | 0% | 25% | 25% | 22% |
| 177 | Lvliang | 15% | 45% | 9% | 0% | 20% | 20% | 11% |
| 178 | Luohe | 15% | 45% | 9% | 0% | 20% | 20% | 11% |
| 179 | Luoyang | 15% | 45% | 9% | 0% | 20% | 20% | 11% |
| 180 | Luzhou | 20% | 18% | 13% | 0% | 19% | 19% | 30% |
| 181 | Loudi | 20% | 18% | 13% | 0% | 19% | 19% | 30% |
| 182 | Longnan | 30% | 30% | 20% | 0% | 20% | 20% | 0% |
| 183 | Longyan | 14% | 31% | 8% | 0% | 25% | 25% | 22% |
| 184 | Liupanshui | 20% | 18% | 13% | 0% | 19% | 19% | 30% |
| 185 | Lu'an | 14% | 31% | 8% | 0% | 25% | 25% | 22% |
| 186 | Liuzhou | 20% | 18% | 13% | 0% | 19% | 19% | 30% |
| 187 | Linyi | 15% | 45% | 9% | 0% | 20% | 20% | 11% |
| 188 | Linxia | 30% | 30% | 20% | 0% | 20% | 20% | 0% |
| 189 | Linfen | 15% | 45% | 9% | 0% | 20% | 20% | 11% |
| 190 | Lincang | 20% | 18% | 13% | 0% | 19% | 19% | 30% |
| 191 | Linzhi | 30% | 30% | 20% | 0% | 20% | 20% | 0% |
| 192 | Liaocheng | 15% | 45% | 9% | 0% | 20% | 20% | 11% |
| 193 | Liaoyuan | 11% | 50% | 28% | 0% | 10% | 10% | 1% |
| 194 | Liaoyang | 11% | 50% | 28% | 0% | 10% | 10% | 1% |
| 195 | Liangshan | 20% | 18% | 13% | 0% | 19% | 19% | 30% |
| 196 | Lianyungang | 14% | 31% | 8% | 0% | 25% | 25% | 22% |
| 197 | Lishui | 14% | 31% | 8% | 0% | 25% | 25% | 22% |
| 198 | Lijiang | 20% | 18% | 13% | 0% | 19% | 19% | 30% |
| 199 | Leshan | 20% | 18% | 13% | 0% | 19% | 19% | 30% |

| | | | | | | | | |
|---|---|---|---|---|---|---|---|---|
| 200 | Langfang | 15% | 45% | 9% | 0% | 20% | 20% | 11% |
| 201 | Lanzhou | 30% | 30% | 20% | 0% | 20% | 20% | 0% |
| 202 | Laiwu | 15% | 45% | 9% | 0% | 20% | 20% | 11% |
| 203 | Laibin | 20% | 18% | 13% | 0% | 19% | 19% | 30% |
| 204 | Lhasa | 30% | 30% | 20% | 0% | 20% | 20% | 0% |
| 205 | Kunming | 20% | 18% | 13% | 0% | 19% | 19% | 30% |
| 206 | Kizilsu Kirgiz | 30% | 30% | 20% | 0% | 20% | 20% | 0% |
| 207 | Qaramay | 30% | 30% | 20% | 0% | 20% | 20% | 0% |
| 208 | Kaifeng | 15% | 45% | 9% | 0% | 20% | 20% | 11% |
| 209 | Kashgar | 30% | 30% | 20% | 0% | 20% | 20% | 0% |
| 210 | Jiuquan | 30% | 30% | 20% | 0% | 20% | 20% | 0% |
| 211 | Jiujiang | 20% | 18% | 13% | 0% | 19% | 19% | 30% |
| 212 | Jingdezhen | 20% | 18% | 13% | 0% | 19% | 19% | 30% |
| 213 | Jingzhou | 20% | 18% | 13% | 0% | 19% | 19% | 30% |
| 214 | Jingmen | 20% | 18% | 13% | 0% | 19% | 19% | 30% |
| 215 | Jinzhong | 15% | 45% | 9% | 0% | 20% | 20% | 11% |
| 216 | Jincheng | 15% | 45% | 9% | 0% | 20% | 20% | 11% |
| 217 | Jinzhou | 11% | 50% | 28% | 0% | 10% | 10% | 1% |
| 218 | Jinhua | 14% | 31% | 8% | 0% | 25% | 25% | 22% |
| 219 | Jinchang | 30% | 30% | 20% | 0% | 20% | 20% | 0% |
| 220 | Jieyang | 20% | 18% | 13% | 0% | 19% | 19% | 30% |
| 221 | Jiaozuo | 15% | 45% | 9% | 0% | 20% | 20% | 11% |
| 222 | Jiangmen | 20% | 18% | 13% | 0% | 19% | 19% | 30% |
| 223 | Jiayuguan | 30% | 30% | 20% | 0% | 20% | 20% | 0% |
| 224 | Jiaxing | 14% | 31% | 8% | 0% | 25% | 25% | 22% |
| 225 | Jiamusi | 11% | 50% | 28% | 0% | 10% | 10% | 1% |
| 226 | Jiyuan | 15% | 45% | 9% | 0% | 20% | 20% | 11% |
| 227 | Jining | 15% | 45% | 9% | 0% | 20% | 20% | 11% |
| 228 | Jinan | 15% | 45% | 9% | 0% | 20% | 20% | 11% |
| 229 | Jilin | 11% | 50% | 28% | 0% | 10% | 10% | 1% |
| 230 | Ji'an | 20% | 18% | 13% | 0% | 19% | 19% | 30% |
| 231 | Chichy | 11% | 50% | 28% | 0% | 10% | 10% | 1% |
| 232 | Huizhou | 20% | 18% | 13% | 0% | 19% | 19% | 30% |
| 233 | Huangshi | 20% | 18% | 13% | 0% | 19% | 19% | 30% |
| 234 | Huangshan | 14% | 31% | 8% | 0% | 25% | 25% | 22% |
| 235 | Huangnan | 30% | 30% | 20% | 0% | 20% | 20% | 0% |
| 236 | Huanggang | 20% | 18% | 13% | 0% | 19% | 19% | 30% |
| 237 | Huainan | 14% | 31% | 8% | 0% | 25% | 25% | 22% |
| 238 | Huaibei | 14% | 31% | 8% | 0% | 25% | 25% | 22% |
| 239 | Huai'an | 14% | 31% | 8% | 0% | 25% | 25% | 22% |
| 240 | Huaihua | 20% | 18% | 13% | 0% | 19% | 19% | 30% |
| 241 | Huzhou | 14% | 31% | 8% | 0% | 25% | 25% | 22% |
| 242 | Huludao | 11% | 50% | 28% | 0% | 10% | 10% | 1% |

| | | | | | | | | |
|---|---|---|---|---|---|---|---|---|
| 243 | Hulunbuir | 15% | 45% | 9% | 0% | 20% | 20% | 11% |
| 244 | Hohhot | 15% | 45% | 9% | 0% | 20% | 20% | 11% |
| 245 | Honghe Hani and Yi | 20% | 18% | 13% | 0% | 19% | 19% | 30% |
| 246 | Hengyang | 20% | 18% | 13% | 0% | 19% | 19% | 30% |
| 247 | Hengshui | 15% | 45% | 9% | 0% | 20% | 20% | 11% |
| 248 | Heihe | 11% | 50% | 28% | 0% | 10% | 10% | 1% |
| 249 | Hegang | 11% | 50% | 28% | 0% | 10% | 10% | 1% |
| 250 | Hebi | 15% | 45% | 9% | 0% | 20% | 20% | 11% |
| 251 | Hehzou | 20% | 18% | 13% | 0% | 19% | 19% | 30% |
| 252 | Heze | 15% | 45% | 9% | 0% | 20% | 20% | 11% |
| 253 | Heyuan | 20% | 18% | 13% | 0% | 19% | 19% | 30% |
| 254 | Hechi | 20% | 18% | 13% | 0% | 19% | 19% | 30% |
| 255 | Khotan | 30% | 30% | 20% | 0% | 20% | 20% | 0% |
| 256 | Hefei | 14% | 31% | 8% | 0% | 25% | 25% | 22% |
| 257 | Hangzhou | 14% | 31% | 8% | 0% | 25% | 25% | 22% |
| 258 | Hanzhong | 30% | 30% | 20% | 0% | 20% | 20% | 0% |
| 259 | Handan | 15% | 45% | 9% | 0% | 20% | 20% | 11% |
| 260 | Haixi | 30% | 30% | 20% | 0% | 20% | 20% | 0% |
| 261 | Hainan | 30% | 30% | 20% | 0% | 20% | 20% | 0% |
| 262 | Haikou | 20% | 18% | 13% | 0% | 19% | 19% | 30% |
| 263 | Haidong | 30% | 30% | 20% | 0% | 20% | 20% | 0% |
| 264 | Haibei | 30% | 30% | 20% | 0% | 20% | 20% | 0% |
| 265 | Hami | 30% | 30% | 20% | 0% | 20% | 20% | 0% |
| 266 | Harbin | 11% | 50% | 28% | 0% | 10% | 10% | 1% |
| 267 | Gologna | 30% | 30% | 20% | 0% | 20% | 20% | 0% |
| 268 | Guilin | 20% | 18% | 13% | 0% | 19% | 19% | 30% |
| 269 | Guiyang | 20% | 18% | 13% | 0% | 19% | 19% | 30% |
| 270 | Guigang | 20% | 18% | 13% | 0% | 19% | 19% | 30% |
| 271 | Guangzhou | 20% | 18% | 13% | 0% | 19% | 19% | 30% |
| 272 | Guangyuan | 20% | 18% | 13% | 0% | 19% | 19% | 30% |
| 273 | Guang'an | 20% | 18% | 13% | 0% | 19% | 19% | 30% |
| 274 | Guyuan | 30% | 30% | 20% | 0% | 20% | 20% | 0% |
| 275 | Ganzhou | 20% | 18% | 13% | 0% | 19% | 19% | 30% |
| 276 | Garze Tibetan | 20% | 18% | 13% | 0% | 19% | 19% | 30% |
| 277 | Gannan | 30% | 30% | 20% | 0% | 20% | 20% | 0% |
| 278 | Fuyang | 14% | 31% | 8% | 0% | 25% | 25% | 22% |
| 279 | Fuxin | 11% | 50% | 28% | 0% | 10% | 10% | 1% |
| 280 | Fuzhou | 20% | 18% | 13% | 0% | 19% | 19% | 30% |
| 281 | Fushun | 11% | 50% | 28% | 0% | 10% | 10% | 1% |
| 282 | Fuzhou | 14% | 31% | 8% | 0% | 25% | 25% | 22% |
| 283 | Foshan | 20% | 18% | 13% | 0% | 19% | 19% | 30% |
| 284 | Fangchenggang | 20% | 18% | 13% | 0% | 19% | 19% | 30% |

| | | | | | | | | |
|---|---|---|---|---|---|---|---|---|
| 285 | Enshi | 20% | 18% | 13% | 0% | 19% | 19% | 30% |
| 286 | Ezhou | 20% | 18% | 13% | 0% | 19% | 19% | 30% |
| 287 | Ordos | 15% | 45% | 9% | 0% | 20% | 20% | 11% |
| 288 | Dongyin | 15% | 45% | 9% | 0% | 20% | 20% | 11% |
| 289 | Dongguan | 20% | 18% | 13% | 0% | 19% | 19% | 30% |
| 290 | Dongfang | 20% | 18% | 13% | 0% | 19% | 19% | 30% |
| 291 | Dingxi | 30% | 30% | 20% | 0% | 20% | 20% | 0% |
| 292 | Diqing Tibetan | 20% | 18% | 13% | 0% | 19% | 19% | 30% |
| 293 | Dezhou | 15% | 45% | 9% | 0% | 20% | 20% | 11% |
| 294 | Deyang | 20% | 18% | 13% | 0% | 19% | 19% | 30% |
| 295 | Dehong Dai and Jingpo | 20% | 18% | 13% | 0% | 19% | 19% | 30% |
| 296 | Zhanzhou | 20% | 18% | 13% | 0% | 19% | 19% | 30% |
| 297 | Dandong | 11% | 50% | 28% | 0% | 10% | 10% | 1% |
| 298 | Daxing'anling | 11% | 50% | 28% | 0% | 10% | 10% | 1% |
| 299 | Datong | 15% | 45% | 9% | 0% | 20% | 20% | 11% |
| 300 | Daqing | 11% | 50% | 28% | 0% | 10% | 10% | 1% |
| 301 | Dalian | 11% | 50% | 28% | 0% | 10% | 10% | 1% |
| 302 | Dali Bai | 20% | 18% | 13% | 0% | 19% | 19% | 30% |
| 303 | Dazhou | 20% | 18% | 13% | 0% | 19% | 19% | 30% |
| 304 | Chuxiong Yi | 20% | 18% | 13% | 0% | 19% | 19% | 30% |
| 305 | Chuzhou | 14% | 31% | 8% | 0% | 25% | 25% | 22% |
| 306 | Chongzuo | 20% | 18% | 13% | 0% | 19% | 19% | 30% |
| 307 | Chifeng | 15% | 45% | 9% | 0% | 20% | 20% | 11% |
| 308 | Chizhou | 14% | 31% | 8% | 0% | 25% | 25% | 22% |
| 309 | Chengde | 15% | 45% | 9% | 0% | 20% | 20% | 11% |
| 310 | Chengdu | 20% | 18% | 13% | 0% | 19% | 19% | 30% |
| 311 | Chenzhou | 20% | 18% | 13% | 0% | 19% | 19% | 30% |
| 312 | Chaozhou | 20% | 18% | 13% | 0% | 19% | 19% | 30% |
| 313 | Chaoyang | 11% | 50% | 28% | 0% | 10% | 10% | 1% |
| 314 | Changzhou | 14% | 31% | 8% | 0% | 25% | 25% | 22% |
| 315 | Changde | 20% | 18% | 13% | 0% | 19% | 19% | 30% |
| 316 | Changji Hui | 30% | 30% | 20% | 0% | 20% | 20% | 0% |
| 317 | Chamdo | 30% | 30% | 20% | 0% | 20% | 20% | 0% |
| 318 | Cangzhou | 15% | 45% | 9% | 0% | 20% | 20% | 11% |
| 319 | Bortala Mongolian | 30% | 30% | 20% | 0% | 20% | 20% | 0% |
| 320 | Haozhou | 14% | 31% | 8% | 0% | 25% | 25% | 22% |
| 321 | Binzhou | 15% | 45% | 9% | 0% | 20% | 20% | 11% |
| 322 | Bijie | 20% | 18% | 13% | 0% | 19% | 19% | 30% |
| 323 | Benxi | 11% | 50% | 28% | 0% | 10% | 10% | 1% |
| 324 | Beijing | 15% | 45% | 9% | 0% | 20% | 20% | 11% |
| 325 | Beihai | 20% | 18% | 13% | 0% | 19% | 19% | 30% |

| | | | | | | | | |
|---|---|---|---|---|---|---|---|---|
| 326 | Baoshan | 20% | 18% | 13% | 0% | 19% | 19% | 30% |
| 327 | Baoding | 15% | 45% | 9% | 0% | 20% | 20% | 11% |
| 328 | Baoji | 30% | 30% | 20% | 0% | 20% | 20% | 0% |
| 329 | Baotou | 15% | 45% | 9% | 0% | 20% | 20% | 11% |
| 330 | Bengbu | 14% | 31% | 8% | 0% | 25% | 25% | 22% |
| 331 | Baise | 20% | 18% | 13% | 0% | 19% | 19% | 30% |
| 332 | Baiyin | 30% | 30% | 20% | 0% | 20% | 20% | 0% |
| 333 | Baishan | 11% | 50% | 28% | 0% | 10% | 10% | 1% |
| 334 | Baicheng | 11% | 50% | 28% | 0% | 10% | 10% | 1% |
| 335 | Bazhong | 20% | 18% | 13% | 0% | 19% | 19% | 30% |
| 336 | Bayingolin Mongolian | 30% | 30% | 20% | 0% | 20% | 20% | 0% |
| 337 | Bayannur | 15% | 45% | 9% | 0% | 20% | 20% | 11% |
| 338 | Anshan | 11% | 50% | 28% | 0% | 10% | 10% | 1% |
| 339 | Anyang | 15% | 45% | 9% | 0% | 20% | 20% | 11% |
| 340 | Anshun | 20% | 18% | 13% | 0% | 19% | 19% | 30% |
| 341 | Anqing | 14% | 31% | 8% | 0% | 25% | 25% | 22% |
| 342 | Ankang | 30% | 30% | 20% | 0% | 20% | 20% | 0% |
| 343 | Ali | 30% | 30% | 20% | 0% | 20% | 20% | 0% |
| 344 | Altay | 30% | 30% | 20% | 0% | 20% | 20% | 0% |
| 345 | Alxa | 15% | 45% | 9% | 0% | 20% | 20% | 11% |
| 346 | Aksu | 30% | 30% | 20% | 0% | 20% | 20% | 0% |
| 347 | Aba Tibetan and Qiang | 20% | 18% | 13% | 0% | 19% | 19% | 30% |

**Table S1.** Swine manure management system ratio in different regions.

| ID | city | Solid storage | Dry lot | Liquid storage | Daily spread | Anaerobic digestion | Combustion | others |
|---|---|---|---|---|---|---|---|---|
| 1 | Zunyi | 50% | 50% | 0% | 0% | 0% | 0% | 0% |
| 2 | Zigong | 50% | 50% | 0% | 0% | 0% | 0% | 0% |
| 3 | Zibo | 23% | 54% | 1% | 0% | 15% | 15% | 6% |
| 4 | Ziyang | 50% | 50% | 0% | 0% | 0% | 0% | 0% |
| 5 | Zhumadian | 23% | 54% | 1% | 0% | 15% | 15% | 6% |
| 6 | Zhuzhou | 25% | 51% | 4% | 0% | 8% | 8% | 12% |
| 7 | Zhuhai | 25% | 51% | 4% | 0% | 8% | 8% | 12% |
| 8 | Zhoukou | 23% | 54% | 1% | 0% | 15% | 15% | 6% |
| 9 | Zhoushan | 23% | 54% | 1% | 0% | 15% | 15% | 7% |
| 10 | Chongqing | 50% | 50% | 0% | 0% | 0% | 0% | 0% |
| 11 | Zhongwei | 53% | 24% | 3% | 1% | 5% | 5% | 8% |
| 12 | Zhongshan | 25% | 51% | 4% | 0% | 8% | 8% | 12% |
| 13 | Zhengzhou | 23% | 54% | 1% | 0% | 15% | 15% | 6% |
| 14 | Zhenjiang | 23% | 54% | 1% | 0% | 15% | 15% | 7% |

| | | | | | | | | |
|---|---|---|---|---|---|---|---|---|
| 15 | Zhaoqing | 25% | 51% | 4% | 0% | 8% | 8% | 12% |
| 16 | Zhaotong | 50% | 50% | 0% | 0% | 0% | 0% | 0% |
| 17 | Changzhi | 24% | 51% | 10% | 1% | 10% | 10% | 3% |
| 18 | Changsha | 25% | 51% | 4% | 0% | 8% | 8% | 12% |
| 19 | Changchun | 85% | 10% | 0% | 0% | 4% | 4% | 1% |
| 20 | Zhangzhou | 23% | 54% | 1% | 0% | 15% | 15% | 7% |
| 21 | Zhangye | 53% | 24% | 3% | 1% | 5% | 5% | 8% |
| 22 | Zhangjiakou | 24% | 51% | 10% | 1% | 10% | 10% | 3% |
| 23 | Zhangjiajie | 25% | 51% | 4% | 0% | 8% | 8% | 12% |
| 24 | Zhanjiang | 25% | 51% | 4% | 0% | 8% | 8% | 12% |
| 25 | Zaozhuang | 23% | 54% | 1% | 0% | 15% | 15% | 6% |
| 26 | Yuncheng | 24% | 51% | 10% | 1% | 10% | 10% | 3% |
| 27 | Yunfu | 25% | 51% | 4% | 0% | 8% | 8% | 12% |
| 28 | Yueyang | 25% | 51% | 4% | 0% | 8% | 8% | 12% |
| 29 | Yuxi | 50% | 50% | 0% | 0% | 0% | 0% | 0% |
| 30 | Yushu | 53% | 24% | 3% | 1% | 5% | 5% | 8% |
| 31 | Yulin | 25% | 51% | 4% | 0% | 8% | 8% | 12% |
| 32 | Yulin | 53% | 24% | 3% | 1% | 5% | 5% | 8% |
| 33 | Yongzhou | 25% | 51% | 4% | 0% | 8% | 8% | 12% |
| 34 | Yingkou | 69% | 10% | 0% | 0% | 4% | 4% | 17% |
| 35 | Yingtan | 23% | 54% | 1% | 0% | 15% | 15% | 7% |
| 36 | Yinchuan | 53% | 24% | 3% | 1% | 5% | 5% | 8% |
| 37 | Yiyang | 25% | 51% | 4% | 0% | 8% | 8% | 12% |
| 38 | Yichun | 23% | 54% | 1% | 0% | 15% | 15% | 7% |
| 39 | Yichang | 25% | 51% | 4% | 0% | 8% | 8% | 12% |
| 40 | Yibin | 50% | 50% | 0% | 0% | 0% | 0% | 0% |
| 41 | Ili Kazak | 53% | 24% | 3% | 1% | 5% | 5% | 8% |
| 42 | Yichun | 85% | 10% | 0% | 0% | 4% | 4% | 1% |
| 43 | Yangquan | 24% | 51% | 10% | 1% | 10% | 10% | 3% |
| 44 | Yangjiang | 25% | 51% | 4% | 0% | 8% | 8% | 12% |
| 45 | Yangzhou | 23% | 54% | 1% | 0% | 15% | 15% | 7% |
| 46 | Yancheng | 23% | 54% | 1% | 0% | 15% | 15% | 7% |
| 47 | Yanbian Korean | 85% | 10% | 0% | 0% | 4% | 4% | 1% |
| 48 | Yan‘an | 53% | 24% | 3% | 1% | 5% | 5% | 8% |
| 49 | Yantai | 23% | 54% | 1% | 0% | 15% | 15% | 6% |
| 50 | Ya'an | 50% | 50% | 0% | 0% | 0% | 0% | 0% |
| 51 | Xuancheng | 23% | 54% | 1% | 0% | 15% | 15% | 7% |
| 52 | Xuchang | 23% | 54% | 1% | 0% | 15% | 15% | 6% |
| 53 | Xuzhou | 23% | 54% | 1% | 0% | 15% | 15% | 7% |
| 54 | Xingan league | 24% | 51% | 10% | 1% | 10% | 10% | 3% |
| 55 | Xingtai | 24% | 51% | 10% | 1% | 10% | 10% | 3% |
| 56 | Xinyang | 23% | 54% | 1% | 0% | 15% | 15% | 6% |

| | | | | | | | | |
|---|---|---|---|---|---|---|---|---|
| 57 | Xinyu | 23% | 54% | 1% | 0% | 15% | 15% | 7% |
| 58 | Xinxiang | 23% | 54% | 1% | 0% | 15% | 15% | 6% |
| 59 | Xinzhou | 24% | 51% | 10% | 1% | 10% | 10% | 3% |
| 60 | Xiaogan | 25% | 51% | 4% | 0% | 8% | 8% | 12% |
| 61 | Xiangyang | 25% | 51% | 4% | 0% | 8% | 8% | 12% |
| 62 | Xiangxi | 25% | 51% | 4% | 0% | 8% | 8% | 12% |
| 63 | Xiangtan | 25% | 51% | 4% | 0% | 8% | 8% | 12% |
| 64 | Xianyang | 53% | 24% | 3% | 1% | 5% | 5% | 8% |
| 65 | Xianning | 25% | 51% | 4% | 0% | 8% | 8% | 12% |
| 66 | Xiantao | 25% | 51% | 4% | 0% | 8% | 8% | 12% |
| 67 | Xilingol League | 24% | 51% | 10% | 1% | 10% | 10% | 3% |
| 68 | Xishuangbanna Dai | 50% | 50% | 0% | 0% | 0% | 0% | 0% |
| 69 | Xining | 53% | 24% | 3% | 1% | 5% | 5% | 8% |
| 70 | Xi'an | 53% | 24% | 3% | 1% | 5% | 5% | 8% |
| 71 | Wuwei | 53% | 24% | 3% | 1% | 5% | 5% | 8% |
| 72 | Wuhan | 25% | 51% | 4% | 0% | 8% | 8% | 12% |
| 73 | Wuzhishan | 25% | 51% | 4% | 0% | 8% | 8% | 12% |
| 74 | Wuzhou | 25% | 51% | 4% | 0% | 8% | 8% | 12% |
| 75 | Wuzhong | 53% | 24% | 3% | 1% | 5% | 5% | 8% |
| 76 | Wuhu | 23% | 54% | 1% | 0% | 15% | 15% | 7% |
| 77 | Wuxi | 23% | 54% | 1% | 0% | 15% | 15% | 7% |
| 78 | Urumqi | 53% | 24% | 3% | 1% | 5% | 5% | 8% |
| 79 | Ulaanchabu | 24% | 51% | 10% | 1% | 10% | 10% | 3% |
| 80 | Wuhai | 24% | 51% | 10% | 1% | 10% | 10% | 3% |
| 81 | Wenshan | 50% | 50% | 0% | 0% | 0% | 0% | 0% |
| 82 | Wenchang | 25% | 51% | 4% | 0% | 8% | 8% | 12% |
| 83 | Wenzhou | 23% | 54% | 1% | 0% | 15% | 15% | 7% |
| 84 | Weinan | 53% | 24% | 3% | 1% | 5% | 5% | 8% |
| 85 | Weifang | 23% | 54% | 1% | 0% | 15% | 15% | 6% |
| 86 | Weihai | 23% | 54% | 1% | 0% | 15% | 15% | 6% |
| 87 | Wanning | 25% | 51% | 4% | 0% | 8% | 8% | 12% |
| 88 | Turpan | 53% | 24% | 3% | 1% | 5% | 5% | 8% |
| 89 | Tongren | 50% | 50% | 0% | 0% | 0% | 0% | 0% |
| 90 | Tongling | 23% | 54% | 1% | 0% | 15% | 15% | 7% |
| 91 | Tongchuan | 53% | 24% | 3% | 1% | 5% | 5% | 8% |
| 92 | Tongliao | 24% | 51% | 10% | 1% | 10% | 10% | 3% |
| 93 | Tonghua | 85% | 10% | 0% | 0% | 4% | 4% | 1% |
| 94 | Tieling | 69% | 10% | 0% | 0% | 4% | 4% | 17% |
| 95 | Tianshui | 53% | 24% | 3% | 1% | 5% | 5% | 8% |
| 96 | Tianmen | 25% | 51% | 4% | 0% | 8% | 8% | 12% |
| 97 | Tianjin | 24% | 51% | 10% | 1% | 10% | 10% | 3% |

| | | | | | | | | |
|---|---|---|---|---|---|---|---|---|
| 98 | Tangshan | 24% | 51% | 10% | 1% | 10% | 10% | 3% |
| 99 | Taizhou | 23% | 54% | 1% | 0% | 15% | 15% | 7% |
| 100 | Tai'an | 23% | 54% | 1% | 0% | 15% | 15% | 6% |
| 101 | Taiyuan | 24% | 51% | 10% | 1% | 10% | 10% | 3% |
| 102 | Taizhou | 23% | 54% | 1% | 0% | 15% | 15% | 7% |
| 103 | Tacheng | 53% | 24% | 3% | 1% | 5% | 5% | 8% |
| 104 | Suining | 50% | 50% | 0% | 0% | 0% | 0% | 0% |
| 105 | Suizhou | 25% | 51% | 4% | 0% | 8% | 8% | 12% |
| 106 | Suihua | 85% | 10% | 0% | 0% | 4% | 4% | 1% |
| 107 | Suzhou | 23% | 54% | 1% | 0% | 15% | 15% | 7% |
| 108 | Suqian | 23% | 54% | 1% | 0% | 15% | 15% | 7% |
| 109 | Suzhou | 23% | 54% | 1% | 0% | 15% | 15% | 7% |
| 110 | Songyuan | 85% | 10% | 0% | 0% | 4% | 4% | 1% |
| 111 | Siping | 85% | 10% | 0% | 0% | 4% | 4% | 1% |
| 112 | Shuozhou | 24% | 51% | 10% | 1% | 10% | 10% | 3% |
| 113 | Shuangyashan | 85% | 10% | 0% | 0% | 4% | 4% | 1% |
| 114 | Shizuishan | 53% | 24% | 3% | 1% | 5% | 5% | 8% |
| 115 | Shijiazhuang | 24% | 51% | 10% | 1% | 10% | 10% | 3% |
| 116 | Shiyan | 25% | 51% | 4% | 0% | 8% | 8% | 12% |
| 117 | Shenyang | 69% | 10% | 0% | 0% | 4% | 4% | 17% |
| 118 | Shennongjia | 25% | 51% | 4% | 0% | 8% | 8% | 12% |
| 119 | Shenzhen | 25% | 51% | 4% | 0% | 8% | 8% | 12% |
| 120 | Shaoxing | 23% | 54% | 1% | 0% | 15% | 15% | 7% |
| 121 | Shaoyang | 25% | 51% | 4% | 0% | 8% | 8% | 12% |
| 122 | Shaoguan | 25% | 51% | 4% | 0% | 8% | 8% | 12% |
| 123 | Shangrao | 23% | 54% | 1% | 0% | 15% | 15% | 7% |
| 124 | Shanghai | 0% | 100% | 0% | 0% | 0% | 0% | 0% |
| 125 | Shangqiu | 23% | 54% | 1% | 0% | 15% | 15% | 6% |
| 126 | Shangluo | 53% | 24% | 3% | 1% | 5% | 5% | 8% |
| 127 | Shanwei | 25% | 51% | 4% | 0% | 8% | 8% | 12% |
| 128 | Shantou | 25% | 51% | 4% | 0% | 8% | 8% | 12% |
| 129 | Shannan | 0% | 0% | 0% | 0% | 0% | 0% | 0% |
| 130 | Xiamen | 23% | 54% | 1% | 0% | 15% | 15% | 7% |
| 131 | Sanya | 25% | 51% | 4% | 0% | 8% | 8% | 12% |
| 132 | Sanming | 23% | 54% | 1% | 0% | 15% | 15% | 7% |
| 133 | Sanmenxia | 23% | 54% | 1% | 0% | 15% | 15% | 6% |
| 134 | Rizhao | 23% | 54% | 1% | 0% | 15% | 15% | 6% |
| 135 | Shigatse | 0% | 0% | 0% | 0% | 0% | 0% | 0% |
| 136 | Quanzhou | 23% | 54% | 1% | 0% | 15% | 15% | 7% |
| 137 | Quzhou | 23% | 54% | 1% | 0% | 15% | 15% | 7% |
| 138 | Qujing | 50% | 50% | 0% | 0% | 0% | 0% | 0% |
| 139 | Qionghai | 25% | 51% | 4% | 0% | 8% | 8% | 12% |
| 140 | Qingyang | 53% | 24% | 3% | 1% | 5% | 5% | 8% |

| | | | | | | | | |
|---|---|---|---|---|---|---|---|---|
| 141 | Qingyuan | 25% | 51% | 4% | 0% | 8% | 8% | 12% |
| 142 | Qingdao | 23% | 54% | 1% | 0% | 15% | 15% | 6% |
| 143 | Qinhuangdao | 24% | 51% | 10% | 1% | 10% | 10% | 3% |
| 144 | Qinzhou | 25% | 51% | 4% | 0% | 8% | 8% | 12% |
| 145 | Qianxinan | 50% | 50% | 0% | 0% | 0% | 0% | 0% |
| 146 | Qiannan | 50% | 50% | 0% | 0% | 0% | 0% | 0% |
| 147 | Qiandongnan | 50% | 50% | 0% | 0% | 0% | 0% | 0% |
| 148 | Qianjiang | 25% | 51% | 4% | 0% | 8% | 8% | 12% |
| 149 | Qiqihar | 85% | 10% | 0% | 0% | 4% | 4% | 1% |
| 150 | Qitaihe | 85% | 10% | 0% | 0% | 4% | 4% | 1% |
| 151 | Pu'er | 50% | 50% | 0% | 0% | 0% | 0% | 0% |
| 152 | Puyang | 23% | 54% | 1% | 0% | 15% | 15% | 6% |
| 153 | Putian | 23% | 54% | 1% | 0% | 15% | 15% | 7% |
| 154 | Pingxiang | 23% | 54% | 1% | 0% | 15% | 15% | 7% |
| 155 | Pingliang | 53% | 24% | 3% | 1% | 5% | 5% | 8% |
| 156 | Pingdingshan | 23% | 54% | 1% | 0% | 15% | 15% | 6% |
| 157 | Panjin | 69% | 10% | 0% | 0% | 4% | 4% | 17% |
| 158 | Panzhihua | 50% | 50% | 0% | 0% | 0% | 0% | 0% |
| 159 | Nujiang Lisu | 50% | 50% | 0% | 0% | 0% | 0% | 0% |
| 160 | Ningde | 23% | 54% | 1% | 0% | 15% | 15% | 7% |
| 161 | Ningbo | 23% | 54% | 1% | 0% | 15% | 15% | 7% |
| 162 | Neijiang | 50% | 50% | 0% | 0% | 0% | 0% | 0% |
| 163 | Nanyang | 23% | 54% | 1% | 0% | 15% | 15% | 6% |
| 164 | Nantong | 23% | 54% | 1% | 0% | 15% | 15% | 7% |
| 165 | Nanping | 23% | 54% | 1% | 0% | 15% | 15% | 7% |
| 166 | Nanning | 25% | 51% | 4% | 0% | 8% | 8% | 12% |
| 167 | Nanjing | 23% | 54% | 1% | 0% | 15% | 15% | 7% |
| 168 | Nnanchong | 50% | 50% | 0% | 0% | 0% | 0% | 0% |
| 169 | Nanchang | 23% | 54% | 1% | 0% | 15% | 15% | 7% |
| 170 | Nagchu | 0% | 0% | 0% | 0% | 0% | 0% | 0% |
| 171 | Mudanjiang | 85% | 10% | 0% | 0% | 4% | 4% | 1% |
| 172 | Mianyang | 50% | 50% | 0% | 0% | 0% | 0% | 0% |
| 173 | Meizhou | 25% | 51% | 4% | 0% | 8% | 8% | 12% |
| 174 | Meishan | 50% | 50% | 0% | 0% | 0% | 0% | 0% |
| 175 | Maoming | 25% | 51% | 4% | 0% | 8% | 8% | 12% |
| 176 | Ma'anshan | 23% | 54% | 1% | 0% | 15% | 15% | 7% |
| 177 | Lvliang | 24% | 51% | 10% | 1% | 10% | 10% | 3% |
| 178 | Luohe | 23% | 54% | 1% | 0% | 15% | 15% | 6% |
| 179 | Luoyang | 23% | 54% | 1% | 0% | 15% | 15% | 6% |
| 180 | Luzhou | 50% | 50% | 0% | 0% | 0% | 0% | 0% |
| 181 | Loudi | 25% | 51% | 4% | 0% | 8% | 8% | 12% |
| 182 | Longnan | 53% | 24% | 3% | 1% | 5% | 5% | 8% |
| 183 | Longyan | 23% | 54% | 1% | 0% | 15% | 15% | 7% |

| | | | | | | | | |
|---|---|---|---|---|---|---|---|---|
| 184 | Liupanshui | 50% | 50% | 0% | 0% | 0% | 0% | 0% |
| 185 | Lu'an | 23% | 54% | 1% | 0% | 15% | 15% | 7% |
| 186 | Liuzhou | 25% | 51% | 4% | 0% | 8% | 8% | 12% |
| 187 | Linyi | 23% | 54% | 1% | 0% | 15% | 15% | 6% |
| 188 | Linxia | 53% | 24% | 3% | 1% | 5% | 5% | 8% |
| 189 | Linfen | 24% | 51% | 10% | 1% | 10% | 10% | 3% |
| 190 | Lincang | 50% | 50% | 0% | 0% | 0% | 0% | 0% |
| 191 | Linzhi | 0% | 0% | 0% | 0% | 0% | 0% | 0% |
| 192 | Liaocheng | 23% | 54% | 1% | 0% | 15% | 15% | 6% |
| 193 | Liaoyuan | 85% | 10% | 0% | 0% | 4% | 4% | 1% |
| 194 | Liaoyang | 69% | 10% | 0% | 0% | 4% | 4% | 17% |
| 195 | Liangshan | 50% | 50% | 0% | 0% | 0% | 0% | 0% |
| 196 | Lianyungang | 23% | 54% | 1% | 0% | 15% | 15% | 7% |
| 197 | Lishui | 23% | 54% | 1% | 0% | 15% | 15% | 7% |
| 198 | Lijiang | 50% | 50% | 0% | 0% | 0% | 0% | 0% |
| 199 | Leshan | 50% | 50% | 0% | 0% | 0% | 0% | 0% |
| 200 | Langfang | 24% | 51% | 10% | 1% | 10% | 10% | 3% |
| 201 | Lanzhou | 53% | 24% | 3% | 1% | 5% | 5% | 8% |
| 202 | Laiwu | 23% | 54% | 1% | 0% | 15% | 15% | 6% |
| 203 | Laibin | 25% | 51% | 4% | 0% | 8% | 8% | 12% |
| 204 | Lhasa | 0% | 0% | 0% | 0% | 0% | 0% | 0% |
| 205 | Kunming | 50% | 50% | 0% | 0% | 0% | 0% | 0% |
| 206 | Kizilsu Kirgiz | 53% | 24% | 3% | 1% | 5% | 5% | 8% |
| 207 | Qaramay | 53% | 24% | 3% | 1% | 5% | 5% | 8% |
| 208 | Kaifeng | 23% | 54% | 1% | 0% | 15% | 15% | 6% |
| 209 | Kashgar | 53% | 24% | 3% | 1% | 5% | 5% | 8% |
| 210 | Jiuquan | 53% | 24% | 3% | 1% | 5% | 5% | 8% |
| 211 | Jiujiang | 23% | 54% | 1% | 0% | 15% | 15% | 7% |
| 212 | Jingdezhen | 23% | 54% | 1% | 0% | 15% | 15% | 7% |
| 213 | Jingzhou | 25% | 51% | 4% | 0% | 8% | 8% | 12% |
| 214 | Jingmen | 25% | 51% | 4% | 0% | 8% | 8% | 12% |
| 215 | Jinzhong | 24% | 51% | 10% | 1% | 10% | 10% | 3% |
| 216 | Jincheng | 24% | 51% | 10% | 1% | 10% | 10% | 3% |
| 217 | Jinzhou | 69% | 10% | 0% | 0% | 4% | 4% | 17% |
| 218 | Jinhua | 23% | 54% | 1% | 0% | 15% | 15% | 7% |
| 219 | Jinchang | 53% | 24% | 3% | 1% | 5% | 5% | 8% |
| 220 | Jieyang | 25% | 51% | 4% | 0% | 8% | 8% | 12% |
| 221 | Jiaozuo | 23% | 54% | 1% | 0% | 15% | 15% | 6% |
| 222 | Jiangmen | 25% | 51% | 4% | 0% | 8% | 8% | 12% |
| 223 | Jiayuguan | 53% | 24% | 3% | 1% | 5% | 5% | 8% |
| 224 | Jiaxing | 23% | 54% | 1% | 0% | 15% | 15% | 7% |
| 225 | Jiamusi | 85% | 10% | 0% | 0% | 4% | 4% | 1% |
| 226 | Jiyuan | 23% | 54% | 1% | 0% | 15% | 15% | 6% |

| | | | | | | | | |
|---|---|---|---|---|---|---|---|---|
| 227 | Jining | 23% | 54% | 1% | 0% | 15% | 15% | 6% |
| 228 | Jinan | 23% | 54% | 1% | 0% | 15% | 15% | 6% |
| 229 | Jilin | 85% | 10% | 0% | 0% | 4% | 4% | 1% |
| 230 | Ji'an | 23% | 54% | 1% | 0% | 15% | 15% | 7% |
| 231 | Chichy | 85% | 10% | 0% | 0% | 4% | 4% | 1% |
| 232 | Huizhou | 25% | 51% | 4% | 0% | 8% | 8% | 12% |
| 233 | Huangshi | 25% | 51% | 4% | 0% | 8% | 8% | 12% |
| 234 | Huangshan | 23% | 54% | 1% | 0% | 15% | 15% | 7% |
| 235 | Huangnan | 53% | 24% | 3% | 1% | 5% | 5% | 8% |
| 236 | Huanggang | 25% | 51% | 4% | 0% | 8% | 8% | 12% |
| 237 | Huainan | 23% | 54% | 1% | 0% | 15% | 15% | 7% |
| 238 | Huaibei | 23% | 54% | 1% | 0% | 15% | 15% | 7% |
| 239 | Huai'an | 23% | 54% | 1% | 0% | 15% | 15% | 7% |
| 240 | Huaihua | 25% | 51% | 4% | 0% | 8% | 8% | 12% |
| 241 | Huzhou | 23% | 54% | 1% | 0% | 15% | 15% | 7% |
| 242 | Huludao | 69% | 10% | 0% | 0% | 4% | 4% | 17% |
| 243 | Hulunbuir | 24% | 51% | 10% | 1% | 10% | 10% | 3% |
| 244 | Hohhot | 24% | 51% | 10% | 1% | 10% | 10% | 3% |
| 245 | Honghe Hani and Yi | 50% | 50% | 0% | 0% | 0% | 0% | 0% |
| 246 | Hengyang | 25% | 51% | 4% | 0% | 8% | 8% | 12% |
| 247 | Hengshui | 24% | 51% | 10% | 1% | 10% | 10% | 3% |
| 248 | Heihe | 85% | 10% | 0% | 0% | 4% | 4% | 1% |
| 249 | Hegang | 85% | 10% | 0% | 0% | 4% | 4% | 1% |
| 250 | Hebi | 23% | 54% | 1% | 0% | 15% | 15% | 6% |
| 251 | Hehzou | 25% | 51% | 4% | 0% | 8% | 8% | 12% |
| 252 | Heze | 23% | 54% | 1% | 0% | 15% | 15% | 6% |
| 253 | Heyuan | 25% | 51% | 4% | 0% | 8% | 8% | 12% |
| 254 | Hechi | 25% | 51% | 4% | 0% | 8% | 8% | 12% |
| 255 | Khotan | 53% | 24% | 3% | 1% | 5% | 5% | 8% |
| 256 | Hefei | 23% | 54% | 1% | 0% | 15% | 15% | 7% |
| 257 | Hangzhou | 23% | 54% | 1% | 0% | 15% | 15% | 7% |
| 258 | Hanzhong | 53% | 24% | 3% | 1% | 5% | 5% | 8% |
| 259 | Handan | 24% | 51% | 10% | 1% | 10% | 10% | 3% |
| 260 | Haixi | 53% | 24% | 3% | 1% | 5% | 5% | 8% |
| 261 | Hainan | 53% | 24% | 3% | 1% | 5% | 5% | 8% |
| 262 | Haikou | 25% | 51% | 4% | 0% | 8% | 8% | 12% |
| 263 | Haidong | 53% | 24% | 3% | 1% | 5% | 5% | 8% |
| 264 | Haibei | 53% | 24% | 3% | 1% | 5% | 5% | 8% |
| 265 | Hami | 53% | 24% | 3% | 1% | 5% | 5% | 8% |
| 266 | Harbin | 85% | 10% | 0% | 0% | 4% | 4% | 1% |
| 267 | Gologna | 53% | 24% | 3% | 1% | 5% | 5% | 8% |
| 268 | Guilin | 25% | 51% | 4% | 0% | 8% | 8% | 12% |

| | | | | | | | | |
|---|---|---|---|---|---|---|---|---|
| 269 | Guiyang | 50% | 50% | 0% | 0% | 0% | 0% | 0% |
| 270 | Guigang | 25% | 51% | 4% | 0% | 8% | 8% | 12% |
| 271 | Guangzhou | 25% | 51% | 4% | 0% | 8% | 8% | 12% |
| 272 | Guangyuan | 50% | 50% | 0% | 0% | 0% | 0% | 0% |
| 273 | Guang'an | 50% | 50% | 0% | 0% | 0% | 0% | 0% |
| 274 | Guyuan | 53% | 24% | 3% | 1% | 5% | 5% | 8% |
| 275 | Ganzhou | 23% | 54% | 1% | 0% | 15% | 15% | 7% |
| 276 | Garze Tibetan | 50% | 50% | 0% | 0% | 0% | 0% | 0% |
| 277 | Gannan | 53% | 24% | 3% | 1% | 5% | 5% | 8% |
| 278 | Fuyang | 23% | 54% | 1% | 0% | 15% | 15% | 7% |
| 279 | Fuxin | 69% | 10% | 0% | 0% | 4% | 4% | 17% |
| 280 | Fuzhou | 23% | 54% | 1% | 0% | 15% | 15% | 7% |
| 281 | Fushun | 69% | 10% | 0% | 0% | 4% | 4% | 17% |
| 282 | Fuzhou | 23% | 54% | 1% | 0% | 15% | 15% | 7% |
| 283 | Foshan | 25% | 51% | 4% | 0% | 8% | 8% | 12% |
| 284 | Fangchenggang | 25% | 51% | 4% | 0% | 8% | 8% | 12% |
| 285 | Enshi | 25% | 51% | 4% | 0% | 8% | 8% | 12% |
| 286 | Ezhou | 25% | 51% | 4% | 0% | 8% | 8% | 12% |
| 287 | Ordos | 24% | 51% | 10% | 1% | 10% | 10% | 3% |
| 288 | Dongyin | 23% | 54% | 1% | 0% | 15% | 15% | 6% |
| 289 | Dongguan | 25% | 51% | 4% | 0% | 8% | 8% | 12% |
| 290 | Dongfang | 25% | 51% | 4% | 0% | 8% | 8% | 12% |
| 291 | Dingxi | 53% | 24% | 3% | 1% | 5% | 5% | 8% |
| 292 | Diqing Tibetan | 50% | 50% | 0% | 0% | 0% | 0% | 0% |
| 293 | Dezhou | 23% | 54% | 1% | 0% | 15% | 15% | 6% |
| 294 | Deyang | 50% | 50% | 0% | 0% | 0% | 0% | 0% |
| 295 | Dehong Dai and Jingpo | 50% | 50% | 0% | 0% | 0% | 0% | 0% |
| 296 | Zhanzhou | 25% | 51% | 4% | 0% | 8% | 8% | 12% |
| 297 | Dandong | 69% | 10% | 0% | 0% | 4% | 4% | 17% |
| 298 | Daxing'anling | 85% | 10% | 0% | 0% | 4% | 4% | 1% |
| 299 | Datong | 24% | 51% | 10% | 1% | 10% | 10% | 3% |
| 300 | Daqing | 85% | 10% | 0% | 0% | 4% | 4% | 1% |
| 301 | Dalian | 69% | 10% | 0% | 0% | 4% | 4% | 17% |
| 302 | Dali Bai | 50% | 50% | 0% | 0% | 0% | 0% | 0% |
| 303 | Dazhou | 50% | 50% | 0% | 0% | 0% | 0% | 0% |
| 304 | Chuxiong Yi | 50% | 50% | 0% | 0% | 0% | 0% | 0% |
| 305 | Chuzhou | 23% | 54% | 1% | 0% | 15% | 15% | 7% |
| 306 | Chongzuo | 25% | 51% | 4% | 0% | 8% | 8% | 12% |
| 307 | Chifeng | 24% | 51% | 10% | 1% | 10% | 10% | 3% |
| 308 | Chizhou | 23% | 54% | 1% | 0% | 15% | 15% | 7% |
| 309 | Chengde | 24% | 51% | 10% | 1% | 10% | 10% | 3% |
| 310 | Chengdu | 50% | 50% | 0% | 0% | 0% | 0% | 0% |

| | | | | | | | | |
|---|---|---|---|---|---|---|---|---|
| 311 | Chenzhou | 25% | 51% | 4% | 0% | 8% | 8% | 12% |
| 312 | Chaozhou | 25% | 51% | 4% | 0% | 8% | 8% | 12% |
| 313 | Chaoyang | 69% | 10% | 0% | 0% | 4% | 4% | 17% |
| 314 | Changzhou | 23% | 54% | 1% | 0% | 15% | 15% | 7% |
| 315 | Changde | 25% | 51% | 4% | 0% | 8% | 8% | 12% |
| 316 | Changji Hui | 53% | 24% | 3% | 1% | 5% | 5% | 8% |
| 317 | Chamdo | 0% | 0% | 0% | 0% | 0% | 0% | 0% |
| 318 | Cangzhou | 24% | 51% | 10% | 1% | 10% | 10% | 3% |
| 319 | Bortala Mongolian | 53% | 24% | 3% | 1% | 5% | 5% | 8% |
| 320 | Haozhou | 23% | 54% | 1% | 0% | 15% | 15% | 7% |
| 321 | Binzhou | 23% | 54% | 1% | 0% | 15% | 15% | 6% |
| 322 | Bijie | 50% | 50% | 0% | 0% | 0% | 0% | 0% |
| 323 | Benxi | 69% | 10% | 0% | 0% | 4% | 4% | 17% |
| 324 | Beijing | 24% | 51% | 10% | 1% | 10% | 10% | 3% |
| 325 | Beihai | 25% | 51% | 4% | 0% | 8% | 8% | 12% |
| 326 | Baoshan | 50% | 50% | 0% | 0% | 0% | 0% | 0% |
| 327 | Baoding | 24% | 51% | 10% | 1% | 10% | 10% | 3% |
| 328 | Baoji | 53% | 24% | 3% | 1% | 5% | 5% | 8% |
| 329 | Baotou | 24% | 51% | 10% | 1% | 10% | 10% | 3% |
| 330 | Bengbu | 23% | 54% | 1% | 0% | 15% | 15% | 7% |
| 331 | Baise | 25% | 51% | 4% | 0% | 8% | 8% | 12% |
| 332 | Baiyin | 53% | 24% | 3% | 1% | 5% | 5% | 8% |
| 333 | Baishan | 85% | 10% | 0% | 0% | 4% | 4% | 1% |
| 334 | Baicheng | 85% | 10% | 0% | 0% | 4% | 4% | 1% |
| 335 | Bazhong | 50% | 50% | 0% | 0% | 0% | 0% | 0% |
| 336 | Bayingolin Mongolian | 53% | 24% | 3% | 1% | 5% | 5% | 8% |
| 337 | Bayannur | 24% | 51% | 10% | 1% | 10% | 10% | 3% |
| 338 | Anshan | 69% | 10% | 0% | 0% | 4% | 4% | 17% |
| 339 | Anyang | 23% | 54% | 1% | 0% | 15% | 15% | 6% |
| 340 | Anshun | 50% | 50% | 0% | 0% | 0% | 0% | 0% |
| 341 | Anqing | 23% | 54% | 1% | 0% | 15% | 15% | 7% |
| 342 | Ankang | 53% | 24% | 3% | 1% | 5% | 5% | 8% |
| 343 | Ali | 0% | 0% | 0% | 0% | 0% | 0% | 0% |
| 344 | Altay | 53% | 24% | 3% | 1% | 5% | 5% | 8% |
| 345 | Alxa | 24% | 51% | 10% | 1% | 10% | 10% | 3% |
| 346 | Aksu | 53% | 24% | 3% | 1% | 5% | 5% | 8% |
| 347 | Aba Tibetan and Qiang | 50% | 50% | 0% | 0% | 0% | 0% | 0% |

**Table S2.** Dairy cattle manure management system ratio in different regions.

| ID | city | Solid storage | Dry lot | Liquid storage | Daily spread | Anaerobic digestion | Combustion | others |
|---|---|---|---|---|---|---|---|---|
| 1 | Zunyi | 50% | 2% | 0% | 0% | 0% | 0% | 48% |
| 2 | Zigong | 50% | 3% | 0% | 0% | 0% | 0% | 47% |
| 3 | Zibo | 22% | 50% | 5% | 2% | 10% | 10% | 10% |
| 4 | Ziyang | 50% | 3% | 0% | 0% | 0% | 0% | 47% |
| 5 | Zhumadian | 22% | 50% | 5% | 2% | 10% | 10% | 10% |
| 6 | Zhuzhou | 14% | 37% | 5% | 0% | 6% | 6% | 38% |
| 7 | Zhuhai | 14% | 37% | 5% | 0% | 6% | 6% | 38% |
| 8 | Zhoukou | 22% | 50% | 5% | 2% | 10% | 10% | 10% |
| 9 | Zhoushan | 22% | 30% | 3% | 3% | 3% | 3% | 39% |
| 10 | Chongqing | 0% | 50% | 0% | 0% | 0% | 0% | 50% |
| 11 | Zhongwei | 86% | 7% | 3% | 1% | 0% | 0% | 3% |
| 12 | Zhongshan | 14% | 37% | 5% | 0% | 6% | 6% | 38% |
| 13 | Zhengzhou | 22% | 50% | 5% | 2% | 10% | 10% | 10% |
| 14 | Zhenjiang | 22% | 30% | 3% | 3% | 3% | 3% | 39% |
| 15 | Zhaoqing | 14% | 37% | 5% | 0% | 6% | 6% | 38% |
| 16 | Zhaotong | 50% | 3% | 0% | 0% | 0% | 0% | 47% |
| 17 | Changzhi | 23% | 50% | 5% | 1% | 10% | 10% | 10% |
| 18 | Changsha | 14% | 37% | 5% | 0% | 6% | 6% | 38% |
| 19 | Changchun | 28% | 58% | 0% | 0% | 3% | 3% | 9% |
| 20 | Zhangzhou | 22% | 30% | 3% | 3% | 3% | 3% | 39% |
| 21 | Zhangye | 64% | 14% | 3% | 1% | 0% | 0% | 7% |
| 22 | Zhangjiakou | 23% | 50% | 5% | 1% | 10% | 10% | 10% |
| 23 | Zhangjiajie | 14% | 37% | 5% | 0% | 6% | 6% | 38% |
| 24 | Zhanjiang | 14% | 37% | 5% | 0% | 6% | 6% | 38% |
| 25 | Zaozhuang | 22% | 50% | 5% | 2% | 10% | 10% | 10% |
| 26 | Yuncheng | 23% | 50% | 5% | 1% | 10% | 10% | 10% |
| 27 | Yunfu | 14% | 37% | 5% | 0% | 6% | 6% | 38% |
| 28 | Yueyang | 14% | 37% | 5% | 0% | 6% | 6% | 38% |
| 29 | Yuxi | 50% | 3% | 0% | 0% | 0% | 0% | 47% |
| 30 | Yushu | 34% | 1% | 3% | 1% | 0% | 0% | 26% |
| 31 | Yulin | 14% | 37% | 5% | 0% | 6% | 6% | 38% |
| 32 | Yulin | 50% | 3% | 0% | 0% | 0% | 0% | 47% |
| 33 | Yongzhou | 14% | 37% | 5% | 0% | 6% | 6% | 38% |
| 34 | Yingkou | 28% | 58% | 0% | 0% | 3% | 3% | 9% |
| 35 | Yingtan | 14% | 37% | 5% | 0% | 6% | 6% | 38% |
| 36 | Yinchuan | 86% | 7% | 3% | 1% | 0% | 0% | 3% |
| 37 | Yiyang | 14% | 37% | 5% | 0% | 6% | 6% | 38% |
| 38 | Yichun | 14% | 37% | 5% | 0% | 6% | 6% | 38% |
| 39 | Yichang | 14% | 37% | 5% | 0% | 6% | 6% | 38% |
| 40 | Yibin | 50% | 3% | 0% | 0% | 0% | 0% | 47% |
| 41 | Ili Kazak | 48% | 17% | 3% | 1% | 0% | 0% | 16% |
| 42 | Yichun | 28% | 58% | 0% | 0% | 3% | 3% | 9% |

| | | | | | | | | |
|---|---|---|---|---|---|---|---|---|
| 43 | Yangquan | 23% | 50% | 5% | 1% | 10% | 10% | 10% |
| 44 | Yangjiang | 14% | 37% | 5% | 0% | 6% | 6% | 38% |
| 45 | Yangzhou | 22% | 30% | 3% | 3% | 3% | 3% | 39% |
| 46 | Yancheng | 22% | 30% | 3% | 3% | 3% | 3% | 39% |
| 47 | Yanbian Korean | 28% | 58% | 0% | 0% | 3% | 3% | 9% |
| 48 | Yan‘an | 50% | 3% | 0% | 0% | 0% | 0% | 47% |
| 49 | Yantai | 22% | 50% | 5% | 2% | 10% | 10% | 10% |
| 50 | Ya'an | 50% | 3% | 0% | 0% | 0% | 0% | 47% |
| 51 | Xuancheng | 22% | 30% | 3% | 3% | 3% | 3% | 39% |
| 52 | Xuchang | 22% | 50% | 5% | 2% | 10% | 10% | 10% |
| 53 | Xuzhou | 22% | 30% | 3% | 3% | 3% | 3% | 39% |
| 54 | Xingan league | 23% | 50% | 5% | 1% | 10% | 10% | 10% |
| 55 | Xingtai | 23% | 50% | 5% | 1% | 10% | 10% | 10% |
| 56 | Xinyang | 22% | 50% | 5% | 2% | 10% | 10% | 10% |
| 57 | Xinyu | 14% | 37% | 5% | 0% | 6% | 6% | 38% |
| 58 | Xinxiang | 22% | 50% | 5% | 2% | 10% | 10% | 10% |
| 59 | Xinzhou | 23% | 50% | 5% | 1% | 10% | 10% | 10% |
| 60 | Xiaogan | 14% | 37% | 5% | 0% | 6% | 6% | 38% |
| 61 | Xiangyang | 14% | 37% | 5% | 0% | 6% | 6% | 38% |
| 62 | Xiangxi | 14% | 37% | 5% | 0% | 6% | 6% | 38% |
| 63 | Xiangtan | 14% | 37% | 5% | 0% | 6% | 6% | 38% |
| 64 | Xianyang | 50% | 3% | 0% | 0% | 0% | 0% | 47% |
| 65 | Xianning | 14% | 37% | 5% | 0% | 6% | 6% | 38% |
| 66 | Xiantao | 14% | 37% | 5% | 0% | 6% | 6% | 38% |
| 67 | Xilingol League | 23% | 50% | 5% | 1% | 10% | 10% | 10% |
| 68 | Xishuangbanna Dai | 50% | 3% | 0% | 0% | 0% | 0% | 47% |
| 69 | Xining | 34% | 1% | 3% | 1% | 0% | 0% | 26% |
| 70 | Xi'an | 50% | 3% | 0% | 0% | 0% | 0% | 47% |
| 71 | Wuwei | 64% | 14% | 3% | 1% | 0% | 0% | 7% |
| 72 | Wuhan | 14% | 37% | 5% | 0% | 6% | 6% | 38% |
| 73 | Wuzhishan | 14% | 37% | 5% | 0% | 6% | 6% | 38% |
| 74 | Wuzhou | 14% | 37% | 5% | 0% | 6% | 6% | 38% |
| 75 | Wuzhong | 86% | 7% | 3% | 1% | 0% | 0% | 3% |
| 76 | Wuhu | 22% | 30% | 3% | 3% | 3% | 3% | 39% |
| 77 | Wuxi | 22% | 30% | 3% | 3% | 3% | 3% | 39% |
| 78 | Urumqi | 48% | 17% | 3% | 1% | 0% | 0% | 16% |
| 79 | Ulaanchabu | 23% | 50% | 5% | 1% | 10% | 10% | 10% |
| 80 | Wuhai | 23% | 50% | 5% | 1% | 10% | 10% | 10% |
| 81 | Wenshan | 50% | 3% | 0% | 0% | 0% | 0% | 47% |
| 82 | Wenchang | 14% | 37% | 5% | 0% | 6% | 6% | 38% |

| | | | | | | | | |
|---|---|---|---|---|---|---|---|---|
| 83 | Wenzhou | 22% | 30% | 3% | 3% | 3% | 3% | 39% |
| 84 | Weinan | 50% | 3% | 0% | 0% | 0% | 0% | 47% |
| 85 | Weifang | 22% | 50% | 5% | 2% | 10% | 10% | 10% |
| 86 | Weihai | 22% | 50% | 5% | 2% | 10% | 10% | 10% |
| 87 | Wanning | 14% | 37% | 5% | 0% | 6% | 6% | 38% |
| 88 | Turpan | 48% | 17% | 3% | 1% | 0% | 0% | 16% |
| 89 | Tongren | 50% | 2% | 0% | 0% | 0% | 0% | 48% |
| 90 | Tongling | 22% | 30% | 3% | 3% | 3% | 3% | 39% |
| 91 | Tongchuan | 50% | 3% | 0% | 0% | 0% | 0% | 47% |
| 92 | Tongliao | 23% | 50% | 5% | 1% | 10% | 10% | 10% |
| 93 | Tonghua | 28% | 58% | 0% | 0% | 3% | 3% | 9% |
| 94 | Tieling | 28% | 58% | 0% | 0% | 3% | 3% | 9% |
| 95 | Tianshui | 64% | 14% | 3% | 1% | 0% | 0% | 7% |
| 96 | Tianmen | 14% | 37% | 5% | 0% | 6% | 6% | 38% |
| 97 | Tianjin | 23% | 50% | 5% | 1% | 10% | 10% | 10% |
| 98 | Tangshan | 23% | 50% | 5% | 1% | 10% | 10% | 10% |
| 99 | Taizhou | 22% | 30% | 3% | 3% | 3% | 3% | 39% |
| 100 | Tai'an | 22% | 50% | 5% | 2% | 10% | 10% | 10% |
| 101 | Taiyuan | 23% | 50% | 5% | 1% | 10% | 10% | 10% |
| 102 | Taizhou | 22% | 30% | 3% | 3% | 3% | 3% | 39% |
| 103 | Tacheng | 48% | 17% | 3% | 1% | 0% | 0% | 16% |
| 104 | Suining | 50% | 3% | 0% | 0% | 0% | 0% | 47% |
| 105 | Suizhou | 14% | 37% | 5% | 0% | 6% | 6% | 38% |
| 106 | Suihua | 28% | 58% | 0% | 0% | 3% | 3% | 9% |
| 107 | Suzhou | 22% | 30% | 3% | 3% | 3% | 3% | 39% |
| 108 | Suqian | 22% | 30% | 3% | 3% | 3% | 3% | 39% |
| 109 | Suzhou | 22% | 30% | 3% | 3% | 3% | 3% | 39% |
| 110 | Songyuan | 28% | 58% | 0% | 0% | 3% | 3% | 9% |
| 111 | Siping | 28% | 58% | 0% | 0% | 3% | 3% | 9% |
| 112 | Shuozhou | 23% | 50% | 5% | 1% | 10% | 10% | 10% |
| 113 | Shuangyashan | 28% | 58% | 0% | 0% | 3% | 3% | 9% |
| 114 | Shizuishan | 86% | 7% | 3% | 1% | 0% | 0% | 3% |
| 115 | Shijiazhuang | 23% | 50% | 5% | 1% | 10% | 10% | 10% |
| 116 | Shiyan | 14% | 37% | 5% | 0% | 6% | 6% | 38% |
| 117 | Shenyang | 28% | 58% | 0% | 0% | 3% | 3% | 9% |
| 118 | Shennongjia | 14% | 37% | 5% | 0% | 6% | 6% | 38% |
| 119 | Shenzhen | 14% | 37% | 5% | 0% | 6% | 6% | 38% |
| 120 | Shaoxing | 22% | 30% | 3% | 3% | 3% | 3% | 39% |
| 121 | Shaoyang | 14% | 37% | 5% | 0% | 6% | 6% | 38% |
| 122 | Shaoguan | 14% | 37% | 5% | 0% | 6% | 6% | 38% |
| 123 | Shangrao | 14% | 37% | 5% | 0% | 6% | 6% | 38% |
| 124 | Shanghai | 22% | 30% | 3% | 3% | 3% | 3% | 39% |
| 125 | Shangqiu | 22% | 50% | 5% | 2% | 10% | 10% | 10% |

| | | | | | | | | |
|---|---|---|---|---|---|---|---|---|
| 126 | Shangluo | 50% | 3% | 0% | 0% | 0% | 0% | 47% |
| 127 | Shanwei | 14% | 37% | 5% | 0% | 6% | 6% | 38% |
| 128 | Shantou | 14% | 37% | 5% | 0% | 6% | 6% | 38% |
| 129 | Shannan | 0% | 0% | 0% | 0% | 0% | 0% | 0% |
| 130 | Xiamen | 22% | 30% | 3% | 3% | 3% | 3% | 39% |
| 131 | Sanya | 14% | 37% | 5% | 0% | 6% | 6% | 38% |
| 132 | Sanming | 22% | 30% | 3% | 3% | 3% | 3% | 39% |
| 133 | Sanmenxia | 22% | 50% | 5% | 2% | 10% | 10% | 10% |
| 134 | Rizhao | 22% | 50% | 5% | 2% | 10% | 10% | 10% |
| 135 | Shigatse | 0% | 0% | 0% | 0% | 0% | 0% | 0% |
| 136 | Quanzhou | 22% | 30% | 3% | 3% | 3% | 3% | 39% |
| 137 | Quzhou | 22% | 30% | 3% | 3% | 3% | 3% | 39% |
| 138 | Qujing | 50% | 3% | 0% | 0% | 0% | 0% | 47% |
| 139 | Qionghai | 14% | 37% | 5% | 0% | 6% | 6% | 38% |
| 140 | Qingyang | 64% | 14% | 3% | 1% | 0% | 0% | 7% |
| 141 | Qingyuan | 14% | 37% | 5% | 0% | 6% | 6% | 38% |
| 142 | Qingdao | 22% | 50% | 5% | 2% | 10% | 10% | 10% |
| 143 | Qinhuangdao | 23% | 50% | 5% | 1% | 10% | 10% | 10% |
| 144 | Qinzhou | 14% | 37% | 5% | 0% | 6% | 6% | 38% |
| 145 | Qianxinan | 50% | 2% | 0% | 0% | 0% | 0% | 48% |
| 146 | Qiannan | 50% | 2% | 0% | 0% | 0% | 0% | 48% |
| 147 | Qiandongnan | 50% | 2% | 0% | 0% | 0% | 0% | 48% |
| 148 | Qianjiang | 14% | 37% | 5% | 0% | 6% | 6% | 38% |
| 149 | Qiqihar | 28% | 58% | 0% | 0% | 3% | 3% | 9% |
| 150 | Qitaihe | 28% | 58% | 0% | 0% | 3% | 3% | 9% |
| 151 | Pu'er | 50% | 3% | 0% | 0% | 0% | 0% | 47% |
| 152 | Puyang | 22% | 50% | 5% | 2% | 10% | 10% | 10% |
| 153 | Putian | 22% | 30% | 3% | 3% | 3% | 3% | 39% |
| 154 | Pingxiang | 14% | 37% | 5% | 0% | 6% | 6% | 38% |
| 155 | Pingliang | 64% | 14% | 3% | 1% | 0% | 0% | 7% |
| 156 | Pingdingshan | 22% | 50% | 5% | 2% | 10% | 10% | 10% |
| 157 | Panjin | 28% | 58% | 0% | 0% | 3% | 3% | 9% |
| 158 | Panzhihua | 50% | 3% | 0% | 0% | 0% | 0% | 47% |
| 159 | Nujiang Lisu | 50% | 3% | 0% | 0% | 0% | 0% | 47% |
| 160 | Ningde | 22% | 30% | 3% | 3% | 3% | 3% | 39% |
| 161 | Ningbo | 22% | 30% | 3% | 3% | 3% | 3% | 39% |
| 162 | Neijiang | 50% | 3% | 0% | 0% | 0% | 0% | 47% |
| 163 | Nanyang | 22% | 50% | 5% | 2% | 10% | 10% | 10% |
| 164 | Nantong | 22% | 30% | 3% | 3% | 3% | 3% | 39% |
| 165 | Nanping | 22% | 30% | 3% | 3% | 3% | 3% | 39% |
| 166 | Nanning | 14% | 37% | 5% | 0% | 6% | 6% | 38% |
| 167 | Nanjing | 22% | 30% | 3% | 3% | 3% | 3% | 39% |
| 168 | Nnanchong | 50% | 3% | 0% | 0% | 0% | 0% | 47% |

| | | | | | | | | |
|---|---|---|---|---|---|---|---|---|
| 169 | Nanchang | 14% | 37% | 5% | 0% | 6% | 6% | 38% |
| 170 | Nagchu | 0% | 0% | 0% | 0% | 0% | 0% | 0% |
| 171 | Mudanjiang | 28% | 58% | 0% | 0% | 3% | 3% | 9% |
| 172 | Mianyang | 50% | 3% | 0% | 0% | 0% | 0% | 47% |
| 173 | Meizhou | 14% | 37% | 5% | 0% | 6% | 6% | 38% |
| 174 | Meishan | 50% | 3% | 0% | 0% | 0% | 0% | 47% |
| 175 | Maoming | 14% | 37% | 5% | 0% | 6% | 6% | 38% |
| 176 | Ma'anshan | 22% | 30% | 3% | 3% | 3% | 3% | 39% |
| 177 | Lvliang | 23% | 50% | 5% | 1% | 10% | 10% | 10% |
| 178 | Luohe | 22% | 50% | 5% | 2% | 10% | 10% | 10% |
| 179 | Luoyang | 22% | 50% | 5% | 2% | 10% | 10% | 10% |
| 180 | Luzhou | 50% | 3% | 0% | 0% | 0% | 0% | 47% |
| 181 | Loudi | 14% | 37% | 5% | 0% | 6% | 6% | 38% |
| 182 | Longnan | 64% | 14% | 3% | 1% | 0% | 0% | 7% |
| 183 | Longyan | 22% | 30% | 3% | 3% | 3% | 3% | 39% |
| 184 | Liupanshui | 50% | 2% | 0% | 0% | 0% | 0% | 48% |
| 185 | Lu'an | 22% | 30% | 3% | 3% | 3% | 3% | 39% |
| 186 | Liuzhou | 14% | 37% | 5% | 0% | 6% | 6% | 38% |
| 187 | Linyi | 22% | 50% | 5% | 2% | 10% | 10% | 10% |
| 188 | Linxia | 64% | 14% | 3% | 1% | 0% | 0% | 7% |
| 189 | Linfen | 23% | 50% | 5% | 1% | 10% | 10% | 10% |
| 190 | Lincang | 50% | 3% | 0% | 0% | 0% | 0% | 47% |
| 191 | Linzhi | 0% | 0% | 0% | 0% | 0% | 0% | 0% |
| 192 | Liaocheng | 22% | 50% | 5% | 2% | 10% | 10% | 10% |
| 193 | Liaoyuan | 28% | 58% | 0% | 0% | 3% | 3% | 9% |
| 194 | Liaoyang | 28% | 58% | 0% | 0% | 3% | 3% | 9% |
| 195 | Liangshan | 50% | 3% | 0% | 0% | 0% | 0% | 47% |
| 196 | Lianyungang | 22% | 30% | 3% | 3% | 3% | 3% | 39% |
| 197 | Lishui | 22% | 30% | 3% | 3% | 3% | 3% | 39% |
| 198 | Lijiang | 50% | 3% | 0% | 0% | 0% | 0% | 47% |
| 199 | Leshan | 50% | 3% | 0% | 0% | 0% | 0% | 47% |
| 200 | Langfang | 23% | 50% | 5% | 1% | 10% | 10% | 10% |
| 201 | Lanzhou | 64% | 14% | 3% | 1% | 0% | 0% | 7% |
| 202 | Laiwu | 22% | 50% | 5% | 2% | 10% | 10% | 10% |
| 203 | Laibin | 14% | 37% | 5% | 0% | 6% | 6% | 38% |
| 204 | Lhasa | 0% | 0% | 0% | 0% | 0% | 0% | 0% |
| 205 | Kunming | 50% | 3% | 0% | 0% | 0% | 0% | 47% |
| 206 | Kizilsu Kirgiz | 48% | 17% | 3% | 1% | 0% | 0% | 16% |
| 207 | Qaramay | 48% | 17% | 3% | 1% | 0% | 0% | 16% |
| 208 | Kaifeng | 22% | 50% | 5% | 2% | 10% | 10% | 10% |
| 209 | Kashgar | 48% | 17% | 3% | 1% | 0% | 0% | 16% |
| 210 | Jiuquan | 64% | 14% | 3% | 1% | 0% | 0% | 7% |
| 211 | Jiujiang | 14% | 37% | 5% | 0% | 6% | 6% | 38% |

| | | | | | | | | |
|---|---|---|---|---|---|---|---|---|
| 212 | Jingdezhen | 14% | 37% | 5% | 0% | 6% | 6% | 38% |
| 213 | Jingzhou | 14% | 37% | 5% | 0% | 6% | 6% | 38% |
| 214 | Jingmen | 14% | 37% | 5% | 0% | 6% | 6% | 38% |
| 215 | Jinzhong | 23% | 50% | 5% | 1% | 10% | 10% | 10% |
| 216 | Jincheng | 23% | 50% | 5% | 1% | 10% | 10% | 10% |
| 217 | Jinzhou | 28% | 58% | 0% | 0% | 3% | 3% | 9% |
| 218 | Jinhua | 22% | 30% | 3% | 3% | 3% | 3% | 39% |
| 219 | Jinchang | 64% | 14% | 3% | 1% | 0% | 0% | 7% |
| 220 | Jieyang | 14% | 37% | 5% | 0% | 6% | 6% | 38% |
| 221 | Jiaozuo | 22% | 50% | 5% | 2% | 10% | 10% | 10% |
| 222 | Jiangmen | 14% | 37% | 5% | 0% | 6% | 6% | 38% |
| 223 | Jiayuguan | 64% | 14% | 3% | 1% | 0% | 0% | 7% |
| 224 | Jiaxing | 22% | 30% | 3% | 3% | 3% | 3% | 39% |
| 225 | Jiamusi | 28% | 58% | 0% | 0% | 3% | 3% | 9% |
| 226 | Jiyuan | 22% | 50% | 5% | 2% | 10% | 10% | 10% |
| 227 | Jining | 22% | 50% | 5% | 2% | 10% | 10% | 10% |
| 228 | Jinan | 22% | 50% | 5% | 2% | 10% | 10% | 10% |
| 229 | Jilin | 28% | 58% | 0% | 0% | 3% | 3% | 9% |
| 230 | Ji'an | 14% | 37% | 5% | 0% | 6% | 6% | 38% |
| 231 | Chichy | 28% | 58% | 0% | 0% | 3% | 3% | 9% |
| 232 | Huizhou | 14% | 37% | 5% | 0% | 6% | 6% | 38% |
| 233 | Huangshi | 14% | 37% | 5% | 0% | 6% | 6% | 38% |
| 234 | Huangshan | 22% | 30% | 3% | 3% | 3% | 3% | 39% |
| 235 | Huangnan | 34% | 1% | 3% | 1% | 0% | 0% | 26% |
| 236 | Huanggang | 14% | 37% | 5% | 0% | 6% | 6% | 38% |
| 237 | Huainan | 22% | 30% | 3% | 3% | 3% | 3% | 39% |
| 238 | Huaibei | 22% | 30% | 3% | 3% | 3% | 3% | 39% |
| 239 | Huai'an | 22% | 30% | 3% | 3% | 3% | 3% | 39% |
| 240 | Huaihua | 14% | 37% | 5% | 0% | 6% | 6% | 38% |
| 241 | Huzhou | 22% | 30% | 3% | 3% | 3% | 3% | 39% |
| 242 | Huludao | 28% | 58% | 0% | 0% | 3% | 3% | 9% |
| 243 | Hulunbuir | 23% | 50% | 5% | 1% | 10% | 10% | 10% |
| 244 | Hohhot | 23% | 50% | 5% | 1% | 10% | 10% | 10% |
| 245 | Honghe Hani and Yi | 50% | 3% | 0% | 0% | 0% | 0% | 47% |
| 246 | Hengyang | 14% | 37% | 5% | 0% | 6% | 6% | 38% |
| 247 | Hengshui | 23% | 50% | 5% | 1% | 10% | 10% | 10% |
| 248 | Heihe | 28% | 58% | 0% | 0% | 3% | 3% | 9% |
| 249 | Hegang | 28% | 58% | 0% | 0% | 3% | 3% | 9% |
| 250 | Hebi | 22% | 50% | 5% | 2% | 10% | 10% | 10% |
| 251 | Hehzou | 14% | 37% | 5% | 0% | 6% | 6% | 38% |
| 252 | Heze | 22% | 50% | 5% | 2% | 10% | 10% | 10% |
| 253 | Heyuan | 14% | 37% | 5% | 0% | 6% | 6% | 38% |

| | | | | | | | | |
|---|---|---|---|---|---|---|---|---|
| 254 | Hechi | 14% | 37% | 5% | 0% | 6% | 6% | 38% |
| 255 | Khotan | 48% | 17% | 3% | 1% | 0% | 0% | 16% |
| 256 | Hefei | 22% | 30% | 3% | 3% | 3% | 3% | 39% |
| 257 | Hangzhou | 22% | 30% | 3% | 3% | 3% | 3% | 39% |
| 258 | Hanzhong | 50% | 3% | 0% | 0% | 0% | 0% | 47% |
| 259 | Handan | 23% | 50% | 5% | 1% | 10% | 10% | 10% |
| 260 | Haixi | 34% | 1% | 3% | 1% | 0% | 0% | 26% |
| 261 | Hainan | 34% | 1% | 3% | 1% | 0% | 0% | 26% |
| 262 | Haikou | 14% | 37% | 5% | 0% | 6% | 6% | 38% |
| 263 | Haidong | 34% | 1% | 3% | 1% | 0% | 0% | 26% |
| 264 | Haibei | 34% | 1% | 3% | 1% | 0% | 0% | 26% |
| 265 | Hami | 48% | 17% | 3% | 1% | 0% | 0% | 16% |
| 266 | Harbin | 28% | 58% | 0% | 0% | 3% | 3% | 9% |
| 267 | Gologna | 34% | 1% | 3% | 1% | 0% | 0% | 26% |
| 268 | Guilin | 14% | 37% | 5% | 0% | 6% | 6% | 38% |
| 269 | Guiyang | 50% | 2% | 0% | 0% | 0% | 0% | 48% |
| 270 | Guigang | 14% | 37% | 5% | 0% | 6% | 6% | 38% |
| 271 | Guangzhou | 14% | 37% | 5% | 0% | 6% | 6% | 38% |
| 272 | Guangyuan | 50% | 3% | 0% | 0% | 0% | 0% | 47% |
| 273 | Guang'an | 50% | 3% | 0% | 0% | 0% | 0% | 47% |
| 274 | Guyuan | 86% | 7% | 3% | 1% | 0% | 0% | 3% |
| 275 | Ganzhou | 14% | 37% | 5% | 0% | 6% | 6% | 38% |
| 276 | Garze Tibetan | 50% | 3% | 0% | 0% | 0% | 0% | 47% |
| 277 | Gannan | 64% | 14% | 3% | 1% | 0% | 0% | 7% |
| 278 | Fuyang | 22% | 30% | 3% | 3% | 3% | 3% | 39% |
| 279 | Fuxin | 28% | 58% | 0% | 0% | 3% | 3% | 9% |
| 280 | Fuzhou | 14% | 37% | 5% | 0% | 6% | 6% | 38% |
| 281 | Fushun | 28% | 58% | 0% | 0% | 3% | 3% | 9% |
| 282 | Fuzhou | 22% | 30% | 3% | 3% | 3% | 3% | 39% |
| 283 | Foshan | 14% | 37% | 5% | 0% | 6% | 6% | 38% |
| 284 | Fangchenggang | 14% | 37% | 5% | 0% | 6% | 6% | 38% |
| 285 | Enshi | 14% | 37% | 5% | 0% | 6% | 6% | 38% |
| 286 | Ezhou | 14% | 37% | 5% | 0% | 6% | 6% | 38% |
| 287 | Ordos | 23% | 50% | 5% | 1% | 10% | 10% | 10% |
| 288 | Dongyin | 22% | 50% | 5% | 2% | 10% | 10% | 10% |
| 289 | Dongguan | 14% | 37% | 5% | 0% | 6% | 6% | 38% |
| 290 | Dongfang | 14% | 37% | 5% | 0% | 6% | 6% | 38% |
| 291 | Dingxi | 64% | 14% | 3% | 1% | 0% | 0% | 7% |
| 292 | Diqing Tibetan | 50% | 3% | 0% | 0% | 0% | 0% | 47% |
| 293 | Dezhou | 22% | 50% | 5% | 2% | 10% | 10% | 10% |
| 294 | Deyang | 50% | 3% | 0% | 0% | 0% | 0% | 47% |
| 295 | Dehong Dai and Jingpo | 50% | 3% | 0% | 0% | 0% | 0% | 47% |

| | | | | | | | | |
|---|---|---|---|---|---|---|---|---|
| 296 | Zhanzhou | 14% | 37% | 5% | 0% | 6% | 6% | 38% |
| 297 | Dandong | 28% | 58% | 0% | 0% | 3% | 3% | 9% |
| 298 | Daxing'anling | 28% | 58% | 0% | 0% | 3% | 3% | 9% |
| 299 | Datong | 23% | 50% | 5% | 1% | 10% | 10% | 10% |
| 300 | Daqing | 28% | 58% | 0% | 0% | 3% | 3% | 9% |
| 301 | Dalian | 28% | 58% | 0% | 0% | 3% | 3% | 9% |
| 302 | Dali Bai | 50% | 3% | 0% | 0% | 0% | 0% | 47% |
| 303 | Dazhou | 50% | 3% | 0% | 0% | 0% | 0% | 47% |
| 304 | Chuxiong Yi | 50% | 3% | 0% | 0% | 0% | 0% | 47% |
| 305 | Chuzhou | 22% | 30% | 3% | 3% | 3% | 3% | 39% |
| 306 | Chongzuo | 14% | 37% | 5% | 0% | 6% | 6% | 38% |
| 307 | Chifeng | 23% | 50% | 5% | 1% | 10% | 10% | 10% |
| 308 | Chizhou | 22% | 30% | 3% | 3% | 3% | 3% | 39% |
| 309 | Chengde | 23% | 50% | 5% | 1% | 10% | 10% | 10% |
| 310 | Chengdu | 50% | 3% | 0% | 0% | 0% | 0% | 47% |
| 311 | Chenzhou | 14% | 37% | 5% | 0% | 6% | 6% | 38% |
| 312 | Chaozhou | 14% | 37% | 5% | 0% | 6% | 6% | 38% |
| 313 | Chaoyang | 28% | 58% | 0% | 0% | 3% | 3% | 9% |
| 314 | Changzhou | 22% | 30% | 3% | 3% | 3% | 3% | 39% |
| 315 | Changde | 14% | 37% | 5% | 0% | 6% | 6% | 38% |
| 316 | Changji Hui | 48% | 17% | 3% | 1% | 0% | 0% | 16% |
| 317 | Chamdo | 0% | 0% | 0% | 0% | 0% | 0% | 0% |
| 318 | Cangzhou | 23% | 50% | 5% | 1% | 10% | 10% | 10% |
| 319 | Bortala Mongolian | 48% | 17% | 3% | 1% | 0% | 0% | 16% |
| 320 | Haozhou | 22% | 30% | 3% | 3% | 3% | 3% | 39% |
| 321 | Binzhou | 22% | 50% | 5% | 2% | 10% | 10% | 10% |
| 322 | Bijie | 50% | 2% | 0% | 0% | 0% | 0% | 48% |
| 323 | Benxi | 28% | 58% | 0% | 0% | 3% | 3% | 9% |
| 324 | Beijing | 23% | 50% | 5% | 1% | 10% | 10% | 10% |
| 325 | Beihai | 14% | 37% | 5% | 0% | 6% | 6% | 38% |
| 326 | Baoshan | 50% | 3% | 0% | 0% | 0% | 0% | 47% |
| 327 | Baoding | 23% | 50% | 5% | 1% | 10% | 10% | 10% |
| 328 | Baoji | 50% | 3% | 0% | 0% | 0% | 0% | 47% |
| 329 | Baotou | 23% | 50% | 5% | 1% | 10% | 10% | 10% |
| 330 | Bengbu | 22% | 30% | 3% | 3% | 3% | 3% | 39% |
| 331 | Baise | 14% | 37% | 5% | 0% | 6% | 6% | 38% |
| 332 | Baiyin | 64% | 14% | 3% | 1% | 0% | 0% | 7% |
| 333 | Baishan | 28% | 58% | 0% | 0% | 3% | 3% | 9% |
| 334 | Baicheng | 28% | 58% | 0% | 0% | 3% | 3% | 9% |
| 335 | Bazhong | 50% | 3% | 0% | 0% | 0% | 0% | 47% |
| 336 | Bayingolin Mongolian | 48% | 17% | 3% | 1% | 0% | 0% | 16% |

| | | | | | | | | |
|---|---|---|---|---|---|---|---|---|
| 337 | Bayannur | 23% | 50% | 5% | 1% | 10% | 10% | 10% |
| 338 | Anshan | 28% | 58% | 0% | 0% | 3% | 3% | 9% |
| 339 | Anyang | 22% | 50% | 5% | 2% | 10% | 10% | 10% |
| 340 | Anshun | 50% | 2% | 0% | 0% | 0% | 0% | 48% |
| 341 | Anqing | 22% | 30% | 3% | 3% | 3% | 3% | 39% |
| 342 | Ankang | 50% | 3% | 0% | 0% | 0% | 0% | 47% |
| 343 | Ali | 0% | 0% | 0% | 0% | 0% | 0% | 0% |
| 344 | Altay | 48% | 17% | 3% | 1% | 0% | 0% | 16% |
| 345 | Alxa | 23% | 50% | 5% | 1% | 10% | 10% | 10% |
| 346 | Aksu | 48% | 17% | 3% | 1% | 0% | 0% | 16% |
| 347 | Aba Tibetan and Qiang | 50% | 3% | 0% | 0% | 0% | 0% | 47% |

**Table S3.** Non-dairy cattle manure management system ratio in different regions.

| ID | city | Solid storage | Dry lot | Liquid storage | Daily spread | Anaerobic digestion | Combustion | Others |
|---|---|---|---|---|---|---|---|---|
| 1 | Zunyi | 51% | 10% | 0% | 1% | 2% | 2% | 30% |
| 2 | Zigong | 51% | 10% | 0% | 1% | 2% | 2% | 30% |
| 3 | Zibo | 47% | 24% | 0% | 6% | 20% | 20% | 2% |
| 4 | Ziyang | 51% | 10% | 0% | 1% | 2% | 2% | 30% |
| 5 | Zhumadian | 23% | 54% | 1% | 0% | 15% | 15% | 6% |
| 6 | Zhuzhou | 38% | 40% | 0% | 0% | 0% | 0% | 22% |
| 7 | Zhuhai | 38% | 40% | 0% | 0% | 0% | 0% | 22% |
| 8 | Zhoukou | 23% | 54% | 1% | 0% | 15% | 15% | 6% |
| 9 | Zhoushan | 51% | 47% | 0% | 0% | 2% | 2% | 0% |
| 10 | Chongqing | 51% | 10% | 0% | 1% | 2% | 2% | 30% |
| 11 | Zhongwei | 51% | 10% | 0% | 1% | 2% | 2% | 30% |
| 12 | Zhongshan | 38% | 40% | 0% | 0% | 0% | 0% | 22% |
| 13 | Zhengzhou | 23% | 54% | 1% | 0% | 15% | 15% | 6% |
| 14 | Zhenjiang | 51% | 47% | 0% | 0% | 2% | 2% | 0% |
| 15 | Zhaoqing | 38% | 40% | 0% | 0% | 0% | 0% | 22% |
| 16 | Zhaotong | 51% | 10% | 0% | 1% | 2% | 2% | 30% |
| 17 | Changzhi | 47% | 24% | 0% | 6% | 0% | 0% | 22% |
| 18 | Changsha | 38% | 40% | 0% | 0% | 0% | 0% | 22% |
| 19 | Changchun | 78% | 0% | 0% | 11% | 0% | 0% | 11% |
| 20 | Zhangzhou | 51% | 47% | 0% | 0% | 2% | 2% | 0% |
| 21 | Zhangye | 51% | 10% | 0% | 1% | 2% | 2% | 30% |
| 22 | Zhangjiakou | 47% | 24% | 0% | 6% | 0% | 0% | 22% |
| 23 | Zhangjiajie | 38% | 40% | 0% | 0% | 0% | 0% | 22% |
| 24 | Zhanjiang | 38% | 40% | 0% | 0% | 0% | 0% | 22% |
| 25 | Zaozhuang | 47% | 24% | 0% | 6% | 20% | 20% | 2% |
| 26 | Yuncheng | 47% | 24% | 0% | 6% | 0% | 0% | 22% |
| 27 | Yunfu | 38% | 40% | 0% | 0% | 0% | 0% | 22% |

| | | | | | | | | |
|---|---|---|---|---|---|---|---|---|
| 28 | Yueyang | 38% | 40% | 0% | 0% | 0% | 0% | 22% |
| 29 | Yuxi | 51% | 10% | 0% | 1% | 2% | 2% | 30% |
| 30 | Yushu | 51% | 10% | 0% | 1% | 2% | 2% | 30% |
| 31 | Yulin | 38% | 40% | 0% | 0% | 0% | 0% | 22% |
| 32 | Yulin | 51% | 10% | 0% | 1% | 2% | 2% | 30% |
| 33 | Yongzhou | 38% | 40% | 0% | 0% | 0% | 0% | 22% |
| 34 | Yingkou | 78% | 0% | 0% | 11% | 0% | 0% | 11% |
| 35 | Yingtan | 38% | 40% | 0% | 0% | 0% | 0% | 22% |
| 36 | Yinchuan | 51% | 10% | 0% | 1% | 2% | 2% | 30% |
| 37 | Yiyang | 38% | 40% | 0% | 0% | 0% | 0% | 22% |
| 38 | Yichun | 38% | 40% | 0% | 0% | 0% | 0% | 22% |
| 39 | Yichang | 38% | 40% | 0% | 0% | 0% | 0% | 22% |
| 40 | Yibin | 51% | 10% | 0% | 1% | 2% | 2% | 30% |
| 41 | Ili Kazak | 51% | 10% | 0% | 1% | 2% | 2% | 30% |
| 42 | Yichun | 78% | 0% | 0% | 11% | 0% | 0% | 11% |
| 43 | Yangquan | 47% | 24% | 0% | 6% | 0% | 0% | 22% |
| 44 | Yangjiang | 38% | 40% | 0% | 0% | 0% | 0% | 22% |
| 45 | Yangzhou | 51% | 47% | 0% | 0% | 2% | 2% | 0% |
| 46 | Yancheng | 51% | 47% | 0% | 0% | 2% | 2% | 0% |
| 47 | Yanbian Korean | 78% | 0% | 0% | 11% | 0% | 0% | 11% |
| 48 | Yan‘an | 51% | 10% | 0% | 1% | 2% | 2% | 30% |
| 49 | Yantai | 47% | 24% | 0% | 6% | 20% | 20% | 2% |
| 50 | Ya'an | 51% | 10% | 0% | 1% | 2% | 2% | 30% |
| 51 | Xuancheng | 51% | 47% | 0% | 0% | 2% | 2% | 0% |
| 52 | Xuchang | 23% | 54% | 1% | 0% | 15% | 15% | 6% |
| 53 | Xuzhou | 51% | 47% | 0% | 0% | 2% | 2% | 0% |
| 54 | Xingan league | 25% | 24% | 0% | 6% | 0% | 0% | 45% |
| 55 | Xingtai | 47% | 24% | 0% | 6% | 0% | 0% | 22% |
| 56 | Xinyang | 23% | 54% | 1% | 0% | 15% | 15% | 6% |
| 57 | Xinyu | 38% | 40% | 0% | 0% | 0% | 0% | 22% |
| 58 | Xinxiang | 23% | 54% | 1% | 0% | 15% | 15% | 6% |
| 59 | Xinzhou | 47% | 24% | 0% | 6% | 0% | 0% | 22% |
| 60 | Xiaogan | 38% | 40% | 0% | 0% | 0% | 0% | 22% |
| 61 | Xiangyang | 38% | 40% | 0% | 0% | 0% | 0% | 22% |
| 62 | Xiangxi | 38% | 40% | 0% | 0% | 0% | 0% | 22% |
| 63 | Xiangtan | 38% | 40% | 0% | 0% | 0% | 0% | 22% |
| 64 | Xianyang | 51% | 10% | 0% | 1% | 2% | 2% | 30% |
| 65 | Xianning | 38% | 40% | 0% | 0% | 0% | 0% | 22% |
| 66 | Xiantao | 38% | 40% | 0% | 0% | 0% | 0% | 22% |
| 67 | Xilingol League | 25% | 24% | 0% | 6% | 0% | 0% | 45% |
| 68 | Xishuangbanna | 51% | 10% | 0% | 1% | 2% | 2% | 30% |

| | | | | | | | | |
|---|---|---|---|---|---|---|---|---|
| | Dai | | | | | | | |
| 69 | Xining | 51% | 10% | 0% | 1% | 2% | 2% | 30% |
| 70 | Xi'an | 51% | 10% | 0% | 1% | 2% | 2% | 30% |
| 71 | Wuwei | 51% | 10% | 0% | 1% | 2% | 2% | 30% |
| 72 | Wuhan | 38% | 40% | 0% | 0% | 0% | 0% | 22% |
| 73 | Wuzhishan | 38% | 40% | 0% | 0% | 0% | 0% | 22% |
| 74 | Wuzhou | 38% | 40% | 0% | 0% | 0% | 0% | 22% |
| 75 | Wuzhong | 51% | 10% | 0% | 1% | 2% | 2% | 30% |
| 76 | Wuhu | 51% | 47% | 0% | 0% | 2% | 2% | 0% |
| 77 | Wuxi | 51% | 47% | 0% | 0% | 2% | 2% | 0% |
| 78 | Urumqi | 51% | 10% | 0% | 1% | 2% | 2% | 30% |
| 79 | Ulaanchabu | 25% | 24% | 0% | 6% | 0% | 0% | 45% |
| 80 | Wuhai | 25% | 24% | 0% | 6% | 0% | 0% | 45% |
| 81 | Wenshan | 51% | 10% | 0% | 1% | 2% | 2% | 30% |
| 82 | Wenchang | 38% | 40% | 0% | 0% | 0% | 0% | 22% |
| 83 | Wenzhou | 51% | 47% | 0% | 0% | 2% | 2% | 0% |
| 84 | Weinan | 51% | 10% | 0% | 1% | 2% | 2% | 30% |
| 85 | Weifang | 47% | 24% | 0% | 6% | 20% | 20% | 2% |
| 86 | Weihai | 47% | 24% | 0% | 6% | 20% | 20% | 2% |
| 87 | Wanning | 38% | 40% | 0% | 0% | 0% | 0% | 22% |
| 88 | Turpan | 51% | 10% | 0% | 1% | 2% | 2% | 30% |
| 89 | Tongren | 51% | 10% | 0% | 1% | 2% | 2% | 30% |
| 90 | Tongling | 51% | 47% | 0% | 0% | 2% | 2% | 0% |
| 91 | Tongchuan | 51% | 10% | 0% | 1% | 2% | 2% | 30% |
| 92 | Tongliao | 25% | 24% | 0% | 6% | 0% | 0% | 45% |
| 93 | Tonghua | 78% | 0% | 0% | 11% | 0% | 0% | 11% |
| 94 | Tieling | 78% | 0% | 0% | 11% | 0% | 0% | 11% |
| 95 | Tianshui | 51% | 10% | 0% | 1% | 2% | 2% | 30% |
| 96 | Tianmen | 38% | 40% | 0% | 0% | 0% | 0% | 22% |
| 97 | Tianjin | 47% | 24% | 0% | 6% | 0% | 0% | 22% |
| 98 | Tangshan | 47% | 24% | 0% | 6% | 0% | 0% | 22% |
| 99 | Taizhou | 51% | 47% | 0% | 0% | 2% | 2% | 0% |
| 100 | Tai'an | 47% | 24% | 0% | 6% | 20% | 20% | 2% |
| 101 | Taiyuan | 47% | 24% | 0% | 6% | 0% | 0% | 22% |
| 102 | Taizhou | 51% | 47% | 0% | 0% | 2% | 2% | 0% |
| 103 | Tacheng | 51% | 10% | 0% | 1% | 2% | 2% | 30% |
| 104 | Suining | 51% | 10% | 0% | 1% | 2% | 2% | 30% |
| 105 | Suizhou | 38% | 40% | 0% | 0% | 0% | 0% | 22% |
| 106 | Suihua | 78% | 0% | 0% | 11% | 0% | 0% | 11% |
| 107 | Suzhou | 51% | 47% | 0% | 0% | 2% | 2% | 0% |
| 108 | Suqian | 51% | 47% | 0% | 0% | 2% | 2% | 0% |
| 109 | Suzhou | 51% | 47% | 0% | 0% | 2% | 2% | 0% |
| 110 | Songyuan | 78% | 0% | 0% | 11% | 0% | 0% | 11% |

| | | | | | | | | |
|---|---|---|---|---|---|---|---|---|
| 111 | Siping | 78% | 0% | 0% | 11% | 0% | 0% | 11% |
| 112 | Shuozhou | 47% | 24% | 0% | 6% | 0% | 0% | 22% |
| 113 | Shuangyashan | 78% | 0% | 0% | 11% | 0% | 0% | 11% |
| 114 | Shizuishan | 51% | 10% | 0% | 1% | 2% | 2% | 30% |
| 115 | Shijiazhuang | 47% | 24% | 0% | 6% | 0% | 0% | 22% |
| 116 | Shiyan | 38% | 40% | 0% | 0% | 0% | 0% | 22% |
| 117 | Shenyang | 78% | 0% | 0% | 11% | 0% | 0% | 11% |
| 118 | Shennongjia | 38% | 40% | 0% | 0% | 0% | 0% | 22% |
| 119 | Shenzhen | 38% | 40% | 0% | 0% | 0% | 0% | 22% |
| 120 | Shaoxing | 51% | 47% | 0% | 0% | 2% | 2% | 0% |
| 121 | Shaoyang | 38% | 40% | 0% | 0% | 0% | 0% | 22% |
| 122 | Shaoguan | 38% | 40% | 0% | 0% | 0% | 0% | 22% |
| 123 | Shangrao | 38% | 40% | 0% | 0% | 0% | 0% | 22% |
| 124 | Shanghai | 51% | 47% | 0% | 0% | 2% | 2% | 0% |
| 125 | Shangqiu | 23% | 54% | 1% | 0% | 15% | 15% | 6% |
| 126 | Shangluo | 51% | 10% | 0% | 1% | 2% | 2% | 30% |
| 127 | Shanwei | 38% | 40% | 0% | 0% | 0% | 0% | 22% |
| 128 | Shantou | 38% | 40% | 0% | 0% | 0% | 0% | 22% |
| 129 | Shannan | 0% | 0% | 0% | 0% | 0% | 0% | 30% |
| 130 | Xiamen | 51% | 47% | 0% | 0% | 2% | 2% | 0% |
| 131 | Sanya | 38% | 40% | 0% | 0% | 0% | 0% | 22% |
| 132 | Sanming | 51% | 47% | 0% | 0% | 2% | 2% | 0% |
| 133 | Sanmenxia | 23% | 54% | 1% | 0% | 15% | 15% | 6% |
| 134 | Rizhao | 47% | 24% | 0% | 6% | 20% | 20% | 2% |
| 135 | Shigatse | 0% | 0% | 0% | 0% | 0% | 0% | 30% |
| 136 | Quanzhou | 51% | 47% | 0% | 0% | 2% | 2% | 0% |
| 137 | Quzhou | 51% | 47% | 0% | 0% | 2% | 2% | 0% |
| 138 | Qujing | 51% | 10% | 0% | 1% | 2% | 2% | 30% |
| 139 | Qionghai | 38% | 40% | 0% | 0% | 0% | 0% | 22% |
| 140 | Qingyang | 51% | 10% | 0% | 1% | 2% | 2% | 30% |
| 141 | Qingyuan | 38% | 40% | 0% | 0% | 0% | 0% | 22% |
| 142 | Qingdao | 47% | 24% | 0% | 6% | 20% | 20% | 2% |
| 143 | Qinhuangdao | 47% | 24% | 0% | 6% | 0% | 0% | 22% |
| 144 | Qinzhou | 38% | 40% | 0% | 0% | 0% | 0% | 22% |
| 145 | Qianxinan | 51% | 10% | 0% | 1% | 2% | 2% | 30% |
| 146 | Qiannan | 51% | 10% | 0% | 1% | 2% | 2% | 30% |
| 147 | Qiandongnan | 51% | 10% | 0% | 1% | 2% | 2% | 30% |
| 148 | Qianjiang | 38% | 40% | 0% | 0% | 0% | 0% | 22% |
| 149 | Qiqihar | 78% | 0% | 0% | 11% | 0% | 0% | 11% |
| 150 | Qitaihe | 78% | 0% | 0% | 11% | 0% | 0% | 11% |
| 151 | Pu'er | 51% | 10% | 0% | 1% | 2% | 2% | 30% |
| 152 | Puyang | 23% | 54% | 1% | 0% | 15% | 15% | 6% |
| 153 | Putian | 51% | 47% | 0% | 0% | 2% | 2% | 0% |

| | | | | | | | | |
|---|---|---|---|---|---|---|---|---|
| 154 | Pingxiang | 38% | 40% | 0% | 0% | 0% | 0% | 22% |
| 155 | Pingliang | 51% | 10% | 0% | 1% | 2% | 2% | 30% |
| 156 | Pingdingshan | 23% | 54% | 1% | 0% | 15% | 15% | 6% |
| 157 | Panjin | 78% | 0% | 0% | 11% | 0% | 0% | 11% |
| 158 | Panzhihua | 51% | 10% | 0% | 1% | 2% | 2% | 30% |
| 159 | Nujiang Lisu | 51% | 10% | 0% | 1% | 2% | 2% | 30% |
| 160 | Ningde | 51% | 47% | 0% | 0% | 2% | 2% | 0% |
| 161 | Ningbo | 51% | 47% | 0% | 0% | 2% | 2% | 0% |
| 162 | Neijiang | 51% | 10% | 0% | 1% | 2% | 2% | 30% |
| 163 | Nanyang | 23% | 54% | 1% | 0% | 15% | 15% | 6% |
| 164 | Nantong | 51% | 47% | 0% | 0% | 2% | 2% | 0% |
| 165 | Nanping | 51% | 47% | 0% | 0% | 2% | 2% | 0% |
| 166 | Nanning | 38% | 40% | 0% | 0% | 0% | 0% | 22% |
| 167 | Nanjing | 51% | 47% | 0% | 0% | 2% | 2% | 0% |
| 168 | Nnanchong | 51% | 10% | 0% | 1% | 2% | 2% | 30% |
| 169 | Nanchang | 38% | 40% | 0% | 0% | 0% | 0% | 22% |
| 170 | Nagchu | 0% | 0% | 0% | 0% | 0% | 0% | 30% |
| 171 | Mudanjiang | 78% | 0% | 0% | 11% | 0% | 0% | 11% |
| 172 | Mianyang | 51% | 10% | 0% | 1% | 2% | 2% | 30% |
| 173 | Meizhou | 38% | 40% | 0% | 0% | 0% | 0% | 22% |
| 174 | Meishan | 51% | 10% | 0% | 1% | 2% | 2% | 30% |
| 175 | Maoming | 38% | 40% | 0% | 0% | 0% | 0% | 22% |
| 176 | Ma'anshan | 51% | 47% | 0% | 0% | 2% | 2% | 0% |
| 177 | Lvliang | 47% | 24% | 0% | 6% | 0% | 0% | 22% |
| 178 | Luohe | 23% | 54% | 1% | 0% | 15% | 15% | 6% |
| 179 | Luoyang | 23% | 54% | 1% | 0% | 15% | 15% | 6% |
| 180 | Luzhou | 51% | 10% | 0% | 1% | 2% | 2% | 30% |
| 181 | Loudi | 38% | 40% | 0% | 0% | 0% | 0% | 22% |
| 182 | Longnan | 51% | 10% | 0% | 1% | 2% | 2% | 30% |
| 183 | Longyan | 51% | 47% | 0% | 0% | 2% | 2% | 0% |
| 184 | Liupanshui | 51% | 10% | 0% | 1% | 2% | 2% | 30% |
| 185 | Lu'an | 51% | 47% | 0% | 0% | 2% | 2% | 0% |
| 186 | Liuzhou | 38% | 40% | 0% | 0% | 0% | 0% | 22% |
| 187 | Linyi | 47% | 24% | 0% | 6% | 20% | 20% | 2% |
| 188 | Linxia | 51% | 10% | 0% | 1% | 2% | 2% | 30% |
| 189 | Linfen | 47% | 24% | 0% | 6% | 0% | 0% | 22% |
| 190 | Lincang | 51% | 10% | 0% | 1% | 2% | 2% | 30% |
| 191 | Linzhi | 0% | 0% | 0% | 0% | 0% | 0% | 30% |
| 192 | Liaocheng | 47% | 24% | 0% | 6% | 20% | 20% | 2% |
| 193 | Liaoyuan | 78% | 0% | 0% | 11% | 0% | 0% | 11% |
| 194 | Liaoyang | 78% | 0% | 0% | 11% | 0% | 0% | 11% |
| 195 | Liangshan | 51% | 10% | 0% | 1% | 2% | 2% | 30% |
| 196 | Lianyungang | 51% | 47% | 0% | 0% | 2% | 2% | 0% |

| | | | | | | | | |
|---|---|---|---|---|---|---|---|---|
| 197 | Lishui | 51% | 47% | 0% | 0% | 2% | 2% | 0% |
| 198 | Lijiang | 51% | 10% | 0% | 1% | 2% | 2% | 30% |
| 199 | Leshan | 51% | 10% | 0% | 1% | 2% | 2% | 30% |
| 200 | Langfang | 47% | 24% | 0% | 6% | 0% | 0% | 22% |
| 201 | Lanzhou | 51% | 10% | 0% | 1% | 2% | 2% | 30% |
| 202 | Laiwu | 47% | 24% | 0% | 6% | 20% | 20% | 2% |
| 203 | Laibin | 38% | 40% | 0% | 0% | 0% | 0% | 22% |
| 204 | Lhasa | 0% | 0% | 0% | 0% | 0% | 0% | 30% |
| 205 | Kunming | 51% | 10% | 0% | 1% | 2% | 2% | 30% |
| 206 | Kizilsu Kirgiz | 51% | 10% | 0% | 1% | 2% | 2% | 30% |
| 207 | Qaramay | 51% | 10% | 0% | 1% | 2% | 2% | 30% |
| 208 | Kaifeng | 23% | 54% | 1% | 0% | 15% | 15% | 6% |
| 209 | Kashgar | 51% | 10% | 0% | 1% | 2% | 2% | 30% |
| 210 | Jiuquan | 51% | 10% | 0% | 1% | 2% | 2% | 30% |
| 211 | Jiujiang | 38% | 40% | 0% | 0% | 0% | 0% | 22% |
| 212 | Jingdezhen | 38% | 40% | 0% | 0% | 0% | 0% | 22% |
| 213 | Jingzhou | 38% | 40% | 0% | 0% | 0% | 0% | 22% |
| 214 | Jingmen | 38% | 40% | 0% | 0% | 0% | 0% | 22% |
| 215 | Jinzhong | 47% | 24% | 0% | 6% | 0% | 0% | 22% |
| 216 | Jincheng | 47% | 24% | 0% | 6% | 0% | 0% | 22% |
| 217 | Jinzhou | 78% | 0% | 0% | 11% | 0% | 0% | 11% |
| 218 | Jinhua | 51% | 47% | 0% | 0% | 2% | 2% | 0% |
| 219 | Jinchang | 51% | 10% | 0% | 1% | 2% | 2% | 30% |
| 220 | Jieyang | 38% | 40% | 0% | 0% | 0% | 0% | 22% |
| 221 | Jiaozuo | 23% | 54% | 1% | 0% | 15% | 15% | 6% |
| 222 | Jiangmen | 38% | 40% | 0% | 0% | 0% | 0% | 22% |
| 223 | Jiayuguan | 51% | 10% | 0% | 1% | 2% | 2% | 30% |
| 224 | Jiaxing | 51% | 47% | 0% | 0% | 2% | 2% | 0% |
| 225 | Jiamusi | 78% | 0% | 0% | 11% | 0% | 0% | 11% |
| 226 | Jiyuan | 23% | 54% | 1% | 0% | 15% | 15% | 6% |
| 227 | Jining | 47% | 24% | 0% | 6% | 20% | 20% | 2% |
| 228 | Jinan | 47% | 24% | 0% | 6% | 20% | 20% | 2% |
| 229 | Jilin | 78% | 0% | 0% | 11% | 0% | 0% | 11% |
| 230 | Ji'an | 38% | 40% | 0% | 0% | 0% | 0% | 22% |
| 231 | Chichy | 78% | 0% | 0% | 11% | 0% | 0% | 11% |
| 232 | Huizhou | 38% | 40% | 0% | 0% | 0% | 0% | 22% |
| 233 | Huangshi | 38% | 40% | 0% | 0% | 0% | 0% | 22% |
| 234 | Huangshan | 51% | 47% | 0% | 0% | 2% | 2% | 0% |
| 235 | Huangnan | 51% | 10% | 0% | 1% | 2% | 2% | 30% |
| 236 | Huanggang | 38% | 40% | 0% | 0% | 0% | 0% | 22% |
| 237 | Huainan | 51% | 47% | 0% | 0% | 2% | 2% | 0% |
| 238 | Huaibei | 51% | 47% | 0% | 0% | 2% | 2% | 0% |
| 239 | Huai'an | 51% | 47% | 0% | 0% | 2% | 2% | 0% |

| | | | | | | | | |
|---|---|---|---|---|---|---|---|---|
| 240 | Huaihua | 38% | 40% | 0% | 0% | 0% | 0% | 22% |
| 241 | Huzhou | 51% | 47% | 0% | 0% | 2% | 2% | 0% |
| 242 | Huludao | 78% | 0% | 0% | 11% | 0% | 0% | 11% |
| 243 | Hulunbuir | 25% | 24% | 0% | 6% | 0% | 0% | 45% |
| 244 | Hohhot | 25% | 24% | 0% | 6% | 0% | 0% | 45% |
| 245 | Honghe Hani and Yi | 51% | 10% | 0% | 1% | 2% | 2% | 30% |
| 246 | Hengyang | 38% | 40% | 0% | 0% | 0% | 0% | 22% |
| 247 | Hengshui | 47% | 24% | 0% | 6% | 0% | 0% | 22% |
| 248 | Heihe | 78% | 0% | 0% | 11% | 0% | 0% | 11% |
| 249 | Hegang | 78% | 0% | 0% | 11% | 0% | 0% | 11% |
| 250 | Hebi | 23% | 54% | 1% | 0% | 15% | 15% | 6% |
| 251 | Hehzou | 38% | 40% | 0% | 0% | 0% | 0% | 22% |
| 252 | Heze | 47% | 24% | 0% | 6% | 20% | 20% | 2% |
| 253 | Heyuan | 38% | 40% | 0% | 0% | 0% | 0% | 22% |
| 254 | Hechi | 38% | 40% | 0% | 0% | 0% | 0% | 22% |
| 255 | Khotan | 51% | 10% | 0% | 1% | 2% | 2% | 30% |
| 256 | Hefei | 51% | 47% | 0% | 0% | 2% | 2% | 0% |
| 257 | Hangzhou | 51% | 47% | 0% | 0% | 2% | 2% | 0% |
| 258 | Hanzhong | 51% | 10% | 0% | 1% | 2% | 2% | 30% |
| 259 | Handan | 47% | 24% | 0% | 6% | 0% | 0% | 22% |
| 260 | Haixi | 51% | 10% | 0% | 1% | 2% | 2% | 30% |
| 261 | Hainan | 51% | 10% | 0% | 1% | 2% | 2% | 30% |
| 262 | Haikou | 38% | 40% | 0% | 0% | 0% | 0% | 22% |
| 263 | Haidong | 51% | 10% | 0% | 1% | 2% | 2% | 30% |
| 264 | Haibei | 51% | 10% | 0% | 1% | 2% | 2% | 30% |
| 265 | Hami | 51% | 10% | 0% | 1% | 2% | 2% | 30% |
| 266 | Harbin | 78% | 0% | 0% | 11% | 0% | 0% | 11% |
| 267 | Gologna | 51% | 10% | 0% | 1% | 2% | 2% | 30% |
| 268 | Guilin | 38% | 40% | 0% | 0% | 0% | 0% | 22% |
| 269 | Guiyang | 51% | 10% | 0% | 1% | 2% | 2% | 30% |
| 270 | Guigang | 38% | 40% | 0% | 0% | 0% | 0% | 22% |
| 271 | Guangzhou | 38% | 40% | 0% | 0% | 0% | 0% | 22% |
| 272 | Guangyuan | 51% | 10% | 0% | 1% | 2% | 2% | 30% |
| 273 | Guang'an | 51% | 10% | 0% | 1% | 2% | 2% | 30% |
| 274 | Guyuan | 51% | 10% | 0% | 1% | 2% | 2% | 30% |
| 275 | Ganzhou | 38% | 40% | 0% | 0% | 0% | 0% | 22% |
| 276 | Garze Tibetan | 51% | 10% | 0% | 1% | 2% | 2% | 30% |
| 277 | Gannan | 51% | 10% | 0% | 1% | 2% | 2% | 30% |
| 278 | Fuyang | 51% | 47% | 0% | 0% | 2% | 2% | 0% |
| 279 | Fuxin | 78% | 0% | 0% | 11% | 0% | 0% | 11% |
| 280 | Fuzhou | 38% | 40% | 0% | 0% | 0% | 0% | 22% |
| 281 | Fushun | 78% | 0% | 0% | 11% | 0% | 0% | 11% |

| | | | | | | | | |
|---|---|---|---|---|---|---|---|---|
| 282 | Fuzhou | 51% | 47% | 0% | 0% | 2% | 2% | 0% |
| 283 | Foshan | 38% | 40% | 0% | 0% | 0% | 0% | 22% |
| 284 | Fangchenggang | 38% | 40% | 0% | 0% | 0% | 0% | 22% |
| 285 | Enshi | 38% | 40% | 0% | 0% | 0% | 0% | 22% |
| 286 | Ezhou | 38% | 40% | 0% | 0% | 0% | 0% | 22% |
| 287 | Ordos | 25% | 24% | 0% | 6% | 0% | 0% | 45% |
| 288 | Dongyin | 47% | 24% | 0% | 6% | 20% | 20% | 2% |
| 289 | Dongguan | 38% | 40% | 0% | 0% | 0% | 0% | 22% |
| 290 | Dongfang | 38% | 40% | 0% | 0% | 0% | 0% | 22% |
| 291 | Dingxi | 51% | 10% | 0% | 1% | 2% | 2% | 30% |
| 292 | Diqing Tibetan | 51% | 10% | 0% | 1% | 2% | 2% | 30% |
| 293 | Dezhou | 47% | 24% | 0% | 6% | 20% | 20% | 2% |
| 294 | Deyang | 51% | 10% | 0% | 1% | 2% | 2% | 30% |
| 295 | Dehong Dai and Jingpo | 51% | 10% | 0% | 1% | 2% | 2% | 30% |
| 296 | Zhanzhou | 38% | 40% | 0% | 0% | 0% | 0% | 22% |
| 297 | Dandong | 78% | 0% | 0% | 11% | 0% | 0% | 11% |
| 298 | Daxing'anling | 78% | 0% | 0% | 11% | 0% | 0% | 11% |
| 299 | Datong | 47% | 24% | 0% | 6% | 0% | 0% | 22% |
| 300 | Daqing | 78% | 0% | 0% | 11% | 0% | 0% | 11% |
| 301 | Dalian | 78% | 0% | 0% | 11% | 0% | 0% | 11% |
| 302 | Dali Bai | 51% | 10% | 0% | 1% | 2% | 2% | 30% |
| 303 | Dazhou | 51% | 10% | 0% | 1% | 2% | 2% | 30% |
| 304 | Chuxiong Yi | 51% | 10% | 0% | 1% | 2% | 2% | 30% |
| 305 | Chuzhou | 51% | 47% | 0% | 0% | 2% | 2% | 0% |
| 306 | Chongzuo | 38% | 40% | 0% | 0% | 0% | 0% | 22% |
| 307 | Chifeng | 25% | 24% | 0% | 6% | 0% | 0% | 45% |
| 308 | Chizhou | 51% | 47% | 0% | 0% | 2% | 2% | 0% |
| 309 | Chengde | 47% | 24% | 0% | 6% | 0% | 0% | 22% |
| 310 | Chengdu | 51% | 10% | 0% | 1% | 2% | 2% | 30% |
| 311 | Chenzhou | 38% | 40% | 0% | 0% | 0% | 0% | 22% |
| 312 | Chaozhou | 38% | 40% | 0% | 0% | 0% | 0% | 22% |
| 313 | Chaoyang | 78% | 0% | 0% | 11% | 0% | 0% | 11% |
| 314 | Changzhou | 51% | 47% | 0% | 0% | 2% | 2% | 0% |
| 315 | Changde | 38% | 40% | 0% | 0% | 0% | 0% | 22% |
| 316 | Changji Hui | 51% | 10% | 0% | 1% | 2% | 2% | 30% |
| 317 | Chamdo | 0% | 0% | 0% | 0% | 0% | 0% | 30% |
| 318 | Cangzhou | 47% | 24% | 0% | 6% | 0% | 0% | 22% |
| 319 | Bortala Mongolian | 51% | 10% | 0% | 1% | 2% | 2% | 30% |
| 320 | Haozhou | 51% | 47% | 0% | 0% | 2% | 2% | 0% |
| 321 | Binzhou | 47% | 24% | 0% | 6% | 20% | 20% | 2% |
| 322 | Bijie | 51% | 10% | 0% | 1% | 2% | 2% | 30% |

| | | | | | | | | |
|---|---|---|---|---|---|---|---|---|
| 323 | Benxi | 78% | 0% | 0% | 11% | 0% | 0% | 11% |
| 324 | Beijing | 47% | 24% | 0% | 6% | 0% | 0% | 22% |
| 325 | Beihai | 38% | 40% | 0% | 0% | 0% | 0% | 22% |
| 326 | Baoshan | 51% | 10% | 0% | 1% | 2% | 2% | 30% |
| 327 | Baoding | 47% | 24% | 0% | 6% | 0% | 0% | 22% |
| 328 | Baoji | 51% | 10% | 0% | 1% | 2% | 2% | 30% |
| 329 | Baotou | 25% | 24% | 0% | 6% | 0% | 0% | 45% |
| 330 | Bengbu | 51% | 47% | 0% | 0% | 2% | 2% | 0% |
| 331 | Baise | 38% | 40% | 0% | 0% | 0% | 0% | 22% |
| 332 | Baiyin | 51% | 10% | 0% | 1% | 2% | 2% | 30% |
| 333 | Baishan | 78% | 0% | 0% | 11% | 0% | 0% | 11% |
| 334 | Baicheng | 78% | 0% | 0% | 11% | 0% | 0% | 11% |
| 335 | Bazhong | 51% | 10% | 0% | 1% | 2% | 2% | 30% |
| 336 | Bayingolin Mongolian | 51% | 10% | 0% | 1% | 2% | 2% | 30% |
| 337 | Bayannur | 25% | 24% | 0% | 6% | 0% | 0% | 45% |
| 338 | Anshan | 78% | 0% | 0% | 11% | 0% | 0% | 11% |
| 339 | Anyang | 23% | 54% | 1% | 0% | 15% | 15% | 6% |
| 340 | Anshun | 51% | 10% | 0% | 1% | 2% | 2% | 30% |
| 341 | Anqing | 51% | 47% | 0% | 0% | 2% | 2% | 0% |
| 342 | Ankang | 51% | 10% | 0% | 1% | 2% | 2% | 30% |
| 343 | Ali | 0% | 0% | 0% | 0% | 0% | 0% | 30% |
| 344 | Altay | 51% | 10% | 0% | 1% | 2% | 2% | 30% |
| 345 | Alxa | 25% | 24% | 0% | 6% | 0% | 0% | 45% |
| 346 | Aksu | 51% | 10% | 0% | 1% | 2% | 2% | 30% |
| 347 | Aba Tibetan and Qiang | 51% | 10% | 0% | 1% | 2% | 2% | 30% |

**Table S4.** Sheep/Goat manure management system ratio in different regions.

| Temperature(℃) | Solid storage | Dry lot | Liquid storage | Daily spread | Anaerobic digestion | Combustion | others |
|---|---|---|---|---|---|---|---|
| ≤10 | 2 | 1 | 10 | 0.5 | 10 | 10 | 1 |
| 11 | 2 | 1 | 11 | 0.5 | 10 | 10 | 1 |
| 12 | 2 | 1 | 13 | 0.5 | 10 | 10 | 1 |
| 13 | 2 | 1 | 14 | 0.5 | 10 | 10 | 1 |
| 14 | 2 | 1 | 15 | 0.5 | 10 | 10 | 1 |
| 15 | 4 | 1 | 17 | 1 | 10 | 10 | 2 |
| 16 | 4 | 2 | 18 | 1 | 10 | 10 | 2 |
| 17 | 4 | 2 | 20 | 1 | 10 | 10 | 2 |
| 18 | 4 | 2 | 22 | 1 | 10 | 10 | 2 |
| 19 | 4 | 2 | 24 | 1 | 10 | 10 | 2 |

| | | | | | | | |
|---|---|---|---|---|---|---|---|
| 20-25 | 4 | 2 | 26 | 1 | 10 | 10 | 2 |
| ≥26 | 5 | 2 | 26 | 1 | 10 | 10 | 2 |

**Table S5.** Methane conversion factors in different mean annual temperature (%).

| Category | ALS_stock_mature | ALS_stock_young | ALS_slaughtered |
|---|---|---|---|
| Dairy cattle | 12 | 6 | 6 |
| Non-dairy cattle | 12 | 6 | 6 |
| Buffalo | 12 | 6 | 6 |
| Sheep | 9 | 2.5 | 5.6 |
| Goat | 9 | 2.5 | 5.6 |
| Swine | 6 | 3 | 3 |
| Horse | 12 | —— | 6 |
| Donkey | 12 | —— | 6 |
| Mule | 12 | —— | 6 |

**Table S6.** Average life span (ALS) of different livestock animals (month).

| Year | Dairy cattle | Non-dairy cattle | Goat | Sheep | Swine | Horse | Donkey | Mule | Poultry | Camel | Rabbit | Buffalo |
|---|---|---|---|---|---|---|---|---|---|---|---|---|
| 2010 | 0.13 | 0.20 | 0.06 | 0.05 | 0.08 | 0.09 | 0.08 | 0.16 | 0.09 | 0.27 | 0.10 | 0.22 |
| 2011 | 0.15 | 0.21 | 0.06 | 0.05 | 0.08 | 0.09 | 0.10 | 0.17 | 0.12 | 0.28 | 0.11 | 0.20 |
| 2012 | 0.16 | 0.22 | 0.06 | 0.06 | 0.09 | 0.11 | 0.12 | 0.18 | 0.13 | 0.21 | 0.08 | 0.30 |
| 2013 | 0.17 | 0.22 | 0.07 | 0.06 | 0.09 | 0.12 | 0.13 | 0.20 | 0.16 | 0.27 | 0.09 | 0.20 |
| 2014 | 0.16 | 0.20 | 0.07 | 0.08 | 0.09 | 0.13 | 0.11 | 0.20 | 0.14 | 0.26 | 0.09 | 0.21 |
| 2015 | 0.17 | 0.19 | 0.07 | 0.06 | 0.10 | 0.12 | 0.12 | 0.21 | 0.11 | 0.38 | 0.08 | 0.22 |
| 2016 | 0.23 | 0.20 | 0.06 | 0.05 | 0.08 | 0.14 | 0.15 | 0.28 | 0.08 | 0.23 | 0.23 | 0.29 |
| 2017 | 0.16 | 0.13 | 0.04 | 0.03 | 0.06 | 0.12 | 0.14 | 0.25 | 0.06 | 0.25 | 0.12 | 0.38 |
| 2018 | 0.11 | 0.11 | 0.01 | 0.02 | 0.03 | 0.05 | 0.05 | 0.15 | 0.01 | 0.29 | 0.10 | 0.43 |
| 2019 | 0.12 | 0.11 | 0.02 | 0.02 | 0.03 | 0.07 | 0.05 | 0.13 | 0.07 | 0.29 | 0.41 | 0.49 |
| 2020 | 0.06 | 0.06 | 0.02 | 0.01 | 0.01 | 0.05 | 0.06 | 0.13 | 0.05 | 0.35 | 0.10 | 0.54 |

**Table S7.** Coefficients of variation of activity data among different dataset (city level, country level and international level).

| Category | Enteric fermentation | Manure management |
|---|---|---|
| Cattles | 25% | 25% |
| Sheep and goats | 25% | 25% |
| Horses | 40% | 25% |
| Donkeys | 40% | 25% |

| | | |
|---|---|---|
| Mules | 40% | 25% |
| Pigs | 40% | 25% |
| Poultry | 40% | 25% |

**Table S8.** Coefficients of variation of EFs of different livestock categories.

| ID | Involving processes | Indicators | Description | Data Sources |
|---|---|---|---|---|
| 1 | Enteric fermentation and manure management | Stocked animal population | The animal population at the end of the year of certain animals in one year. | ● China Animal Husbandry and Veterinary (https://data.cnki.net/yearBook/single?id=N2023030190)<br>● Province statistic yearbook (https://data.cnki.net/)<br>● City statistic yearbook (https://data.cnki.net/)<br>● City statistic bulletin (in the government website, such as: https://www.km.gov.cn/c/2021-06-22/3985015.shtml)<br>● Online official reports |
| 2 | Enteric fermentation and manure management | Slaughtered animal population | The slaughtered animal population within this year of certain animals in one year. | ● China Animal Husbandry and Veterinary (https://data.cnki.net/yearBook/single?id=N2023030190)<br>● Province statistic yearbook (https://data.cnki.net/)<br>● City statistic yearbook (https://data.cnki.net/)<br>● City statistic bulletin (in the government website, such as: https://www.km.gov.cn/c/2021-06-22/3985015.shtml)<br>● Online official reports |
| 3 | Enteric fermentation and manure management | Animal weight | The average body weight of the certain animal categories in certain years (kg). | China Agricultural Products Cost-benefit Information Compilation (CAPCIC) |
| 4 | Enteric fermentation and manure management | Average life span | The months that animals emit methane within a calendar year of livestock category | Zhang, et al. [4] |
| 5 | Enteric fermentation | Wool production | Average wool production one day (kg). | China Agriculture Yearbook (https://data.cnki.net/yearBook/single?id=N2023030189) |
| 6 | Enteric fermentation | Milk production | Average milk production one day (kg). | China Dairy Yearbook (https://data.cnki.net/yearBook/single?id=N2013120207) |
| 8 | Enteric fermentation | The EF of non-ruminant animals | The emission factors of non-ruminant animals. | The Guidelines for Provincial Greenhouse Gas Inventories （Trial） |
| 9 | Enteric fermentation | Other parameters in the | The constant and default coefficients mentioned in the estimation. | 2019 Refinement to the 2006 IPCC Guidelines for National Greenhouse Gas Inventories |

| | | | | |
|---|---|---|---|---|
| | | estimation | | |
| 10 | Manure management | Manure management styles | The fraction of different manure management style in one city's livestock system (%). | The People's Republic of China National Greenhouse Gas Inventory |
| 11 | Manure management | Temperature | The annual temperature of the certain city in ℃. | Province statistic yearbook (https://data.cnki.net/ ) |
| 12 | Manure management | Other parameters in the estimation | The constant and default coefficients mentioned in the estimation. | 2019 Refinement to the 2006 IPCC Guidelines for National Greenhouse Gas Inventories |
| 13 | Uncertainty analysis | Activity data | Stock and slaughtered animal population. | FAOSTSAT(https://www.fao.org/faostat/en/#home) |

**Table S9.** Data sources of the main data in this study.

| Year | This study | 95%_low | 95% _high | Min_interval | Max_interval |
|---|---|---|---|---|---|
| 2010 | 13.04 | 10.29 | 15.37 | -21% | 18% |
| 2011 | 12.98 | 10.25 | 15.28 | -21% | 18% |
| 2012 | 13.14 | 10.36 | 15.55 | -21% | 18% |
| 2013 | 13.45 | 10.65 | 15.77 | -21% | 17% |
| 2014 | 13.42 | 10.64 | 15.74 | -21% | 17% |
| 2015 | 13.57 | 10.74 | 15.91 | -21% | 17% |
| 2016 | 12.78 | 10.07 | 15.01 | -21% | 17% |
| 2017 | 11.50 | 9.08 | 13.62 | -21% | 18% |
| 2018 | 11.21 | 8.84 | 13.20 | -21% | 18% |
| 2019 | 11.05 | 8.64 | 13.08 | -22% | 18% |
| 2020 | 11.51 | 9.56 | 13.07 | -17% | 14% |

**Table S10.** The uncertainty interval of the final emission results.

## Reference


1. IPCC. Intergovernmental panel on climate change (IPCC) Guidelines for National Greenhouse Gas Inventories. (IPCC, 2019).
2. Yu, J., *et al.* Inventory of methane emissions from livestock in China from 1980 to 2013. *Atmospheric Environment* **184**, 69-76 (2018).
3. Dairy Association of China. China Dairy Yearbook. (China Agriculture Press, 2011-2021)
4. Zhang, L., *et al.* Methane emissions from livestock in East Asia during 1961-2019. *Ecosystem Health and Sustainability* **7**(2021).
